# Aqueous-phase secondary organic aerosol formation on mineral dust

**DOI:** 10.1093/nsr/nwaf221

**Published:** 2025-05-31

**Authors:** Weijun Li, Akinori Ito, Guochen Wang, Minkang Zhi, Liang Xu, Qi Yuan, Jian Zhang, Lei Liu, Feng Wu, Alexander Laskin, Daizhou Zhang, Xiaoye Zhang, Tong Zhu, Jianmin Chen, Nikolaos Mihalopoulos, Aikaterini Bougiatioti, Maria Kanakidou, Gehui Wang, Huilin Hu, Yue Zhao, Zongbo Shi

**Affiliations:** State Key Laboratory of Ocean Sensing and Department of Atmospheric Sciences, School of Earth Sciences, Zhejiang University, Hangzhou 310027, China; Yokohama Institute for Earth Sciences, JAMSTEC, Yokohama 236-0001, Japan; State Key Laboratory of Ocean Sensing and Department of Atmospheric Sciences, School of Earth Sciences, Zhejiang University, Hangzhou 310027, China; State Key Laboratory of Ocean Sensing and Department of Atmospheric Sciences, School of Earth Sciences, Zhejiang University, Hangzhou 310027, China; State Key Laboratory of Ocean Sensing and Department of Atmospheric Sciences, School of Earth Sciences, Zhejiang University, Hangzhou 310027, China; State Key Laboratory of Ocean Sensing and Department of Atmospheric Sciences, School of Earth Sciences, Zhejiang University, Hangzhou 310027, China; State Key Laboratory of Ocean Sensing and Department of Atmospheric Sciences, School of Earth Sciences, Zhejiang University, Hangzhou 310027, China; State Key Laboratory of Ocean Sensing and Department of Atmospheric Sciences, School of Earth Sciences, Zhejiang University, Hangzhou 310027, China; Key Laboratory of Aerosol Chemistry & Physics and State Key Laboratory of Loess and Quaternary Geology, Institute of Earth Environment, Chinese Academy of Sciences, Xi'an 710061, China; Department of Chemistry, Purdue University, West Lafayette, IN 47907, USA; Faculty of Environmental and Symbiotic Sciences, Prefectural University of Kumamoto, Kumamoto 862-8502, Japan; Key Laboratory of Atmospheric Chemistry of CMA, Institute of Atmospheric Composition, Chinese Academy of Meteorological Sciences, Beijing 100081, China; College of Environmental Sciences and Engineering, Peking University, Beijing 100871, China; Shanghai Key Laboratory of Atmospheric Particle Pollution and Prevention, Department of Environmental Science and Engineering, Fudan University, Shanghai 200433, China; Institute for Environmental Research and Sustainable Development, National Observatory of Athens, Athens 15236, Greece; Environmental Chemical Processes Laboratory, Department of Chemistry, University of Crete, Heraklion 70013, Greece; Institute for Environmental Research and Sustainable Development, National Observatory of Athens, Athens 15236, Greece; Environmental Chemical Processes Laboratory, Department of Chemistry, University of Crete, Heraklion 70013, Greece; Environmental Chemical Processes Laboratory, Department of Chemistry, University of Crete, Heraklion 70013, Greece; Institute of Environmental Physics, Department of Physics, University of Bremen, Bremen 28359, Germany; Center for the Study of Air Quality and Climate Change (C-STACC), Institute of Chemical Engineering Sciences (ICE-HT), Foundation for Research and Technology, Hellas (FORTH), Patras 26504, Greece; Key Lab of Geographic Information Science of the Ministry of Education, School of Geographic Sciences, East China Normal University, Shanghai 210062, China; School of Environmental Science and Engineering, Shanghai Jiao Tong University, Shanghai 200240, China; School of Environmental Science and Engineering, Shanghai Jiao Tong University, Shanghai 200240, China; School of Geography, Earth and Environmental Sciences, University of Birmingham, Birmingham B15 2TT, UK; Anhui Institute of Optics and Fine Mechanics, Chinese Academy of Sciences, Hefei 231137 China

**Keywords:** secondary organic aerosol, dust particles, hygroscopicity, aqueous-phase reactions

## Abstract

Secondary organic aerosol (SOA) is a significant component of airborne particles that impacts air quality, health, and climate globally. Aqueous-phase reactions contribute substantially to SOA mass. However, this process is primarily treated as occurring in submicron particles that contain water, or within cloud droplets in state-of-the-art models. Here, we challenged this conventional view by showing that >50% of water-soluble organic carbon (WSOC), predominantly SOA, is found in supermicron particles during dust events downwind of Saharan and Asian dust sources. Even on non-dust days, supermicron WSOC contributes 25%–51% of total WSOC. Microscopic analyses revealed that organic matter was only detected on aged dust containing a calcium nitrate coating, which contains water at typical ambient relative humidity conditions. This suggests that it is the water-containing nitrate coating that facilitates aqueous-phase SOA formation. By incorporating the reactive uptake of glyoxal, a key precursor of SOA, into a global model, we significantly improved the model's performance in reproducing supermicron particle contributions to total WSOC observed in the field. Using this improved model, aqueous-phase SOA formed on dust particles over the land contributes to 16% of total SOA and 28% of total aqueous-phase SOA, with levels reaching up to 67% and 74% across the ‘dust belt’, respectively. These results underscore the important role of aqueous-phase reactions in aged nitrate-containing dust in SOA formation, which should be incorporated into global models to quantify their potential implications for air quality, health, and climate.

## Teaser

Aged dust particles serve as a medium for producing approximately 28% of aqueous-phase secondary organic aerosol (aqSOA) on supermicron dust over land.

## INTRODUCTION

Organic aerosols, often dominated by secondary organic aerosols (SOAs), contribute 20%–90% of submicron (<1 µm) aerosol mass [1,2], with global impacts on air quality, clouds, and climate [3–5]. SOA is formed through condensation following atmospheric oxidation of volatile organic compounds (VOCs) [1,6,7] and reactions in the cloud and aerosol water (e.g. the photooxidation of glyoxal) [3,8–11], the latter of which is defined as aqueous-phase SOA (aqSOA). aqSOA has been suggested to contribute substantial mass to submicron SOA [3,8,12,13] and emerging evidence points toward aqSOA as an important missing pathway for SOA formation in models [10,12,14]. Currently, aqSOA is only simulated in a few atmospheric models, in which it is primarily formed in cloud water, and predominantly in the submicron particles [5,8,15,16]. Three atmospheric models incorporate aqSOA in aerosol water, which show a better agreement with SOA observations [17–19]. In these models, aqSOA is primarily formed from glyoxal and methyl-glyoxal, as one of the most abundant water-soluble volatile organic compounds in the atmosphere [20], in cloud water and thus is mainly associated with submicrometer sulfate aerosols [8].

However, aged dust particles, primarily in supermicrons (>1 µm), also contain a substantial amount of water [21–24], which could act as a medium for aqSOA formation [25]. Note that dust has the largest terrestrial source of aerosol particle mass and an atmospheric burden of ∼26 Tg [26], which is more than an order of magnitude higher than that of SOA (∼1.06 Tg) [27]. Since fresh dust particles are usually hydrophobic, it is the secondary species, such as sulfate and nitrate, formed on aged dust particles that absorb water (Table S1) [21,23,24,28,29]. This suggests that aged dust can act as a medium to form aqSOA. Indeed, field observations indicated the presence of oxalate, an important secondary organic species, in aged supermicron particles during dust events, alongside nitrate [30–34]. Moreover, glyoxal photooxidation mediated by nitrate photolysis and photosensitization has the potential to enhance the atmospheric sink of glyoxal to contribute to aqSOA [11]. However, field measurement studies have yet to provide any conclusive evidence on the formation of aqSOA on dust particles, and as a result, no models have considered the potential importance of this process.

Here, we carried out size-resolved aerosol chemistry observations to show that water-soluble organic carbon (WSOC), mostly secondary in origin [14,35], in supermicron mode contributes more than half of the total WSOC during dust events in Alashan, Xi'an, Qingdao, and Crete. We carried out single particle analysis to understand why WSOC is found in the supermicron particles. We further carried out global modelling to investigate the role of aqueous reactions in SOA formation on aged dust.

## RESULTS

### Observational evidence of SOA formation on aged dust particles

**Figure 1.**
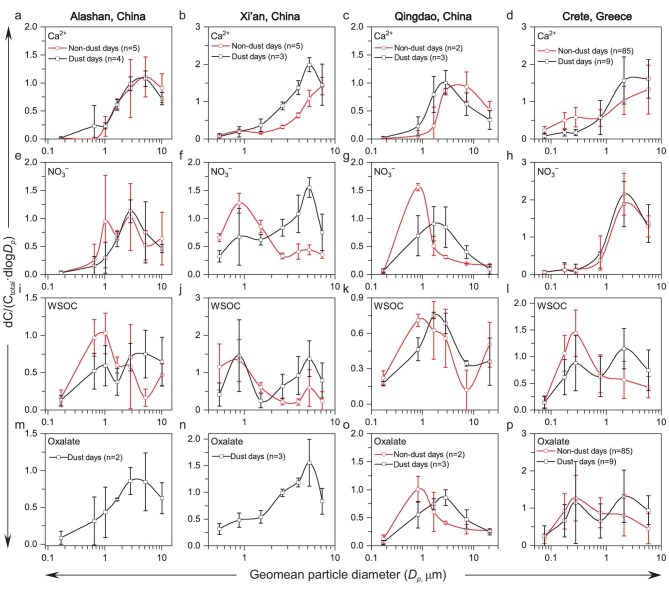
Size distribution of Ca^2+^, NO_3_^−^, WSOC, and oxalate at Alashan (a, e, i, m), Xi'an (b, f, j, n), Qingdao (c, g, k, o), and Crete (d, h, l, p) (see also Fig. S15). Ca^2+^ (a–d) is primarily in the supermicron mode. NO_3_^−^ (e–h) is primarily in the supermicron mode during dust events. WSOC (i–l) is mainly in the sub-micron mode during non-dust periods but has a supermicron mode during dust events. Oxalate (m–p) is primarily in the supermicron particles during dust events and submicron particles during non-dust periods. The above data are normalized based on the dC_i_/dC_i-all_ (see Methods). The center points and bars represent average values from different samples and their standard errors, respectively.

Figure 1 shows the mass size distributions of Ca^2+^, NO_3_^−^, WSOC, and oxalate during dust and non-dust periods at Alashan, Xi'an, Qingdao, and Crete (locations shown in Fig. S1). Ca^2+^ was primarily in the supermicron particles (>1 µm) at all sites and during all sampling periods (Fig. 1a–d). NO_3_^−^ was mainly in submicron mode in Alashan, Xi'an, and Qingdao (Fig. 1e–g) and the supermicron mode in Crete during non-dust periods (Fig. 1). However, NO_3_^−^ was predominantly in supermicron mode in all locations during dust events (Fig. 1e–h, Fig. S1). In comparison, a new nitrate mode in sub-micron sizes appeared in Alashan, Xi'an, and Qingdao that is not associated with Ca²⁺, likely due to the formation of ammonium nitrate. Moreover, size distributions of WSOC are different between dust-influenced and non-dust periods (Fig. 1i–l). The supermicron WSOC peaks are evident during dust events at all locations and correspond to Ca^2+^. There is another WSOC peak in the submicron mode during dust events at Alashan, Xi'an, and Crete, but they do not correspond to Ca^2+^, suggesting a different formation mechanism.

The total mass concentrations of WSOC in supermicron particles during dust events were 10.3 ± 7.6 μg m⁻³ in Alashan, 17.8 ± 10.5 μg m⁻³ in Xi’an, 6.3 ± 2.5 μg m⁻³ in Qingdao, and 0.6 ± 0.4 μg m⁻³ in Crete. In comparison, the WSOC mass loadings during non-dust days were 3.2 ± 2.5 μg m⁻³ in Alashan, 6.8 ± 4.7 μg m⁻³ in Qingdao, and 0.2 ± 0.2 μg m⁻³ in Crete. Except for Qingdao, WSOC concentrations during dust events in Alashan and Crete were ∼2 to 3 times higher than those during non-dust periods. The total mass loadings of WSOC in the submicron particles were 4.4 ± 1.9, 9.5 ± 4.2, 3.3 ± 1.1, and 0.6 ± 0.3 μg m⁻³ during dust days in Alashan, Xi'an, Qingdao, and Crete, respectively. As for non-dust days, the total WSOC in the submicron particles were 3.7 ± 2.8, 6.0 ± 2.6, and 1.3 ± 0.3 μg m⁻³ in Alashan, Qingdao, and Crete, respectively. Overall, the levels of total WSOC in the supermicron particles were significantly higher than those in the submicron particles during dust days. However, except for Qingdao, WSOC concentrations in the supermicron particles on non-dust days were lower than those in the submicron particles in Alashan and Crete. Overall, WSOC in supermicron particles accounted for 66% ± 14%, 61% ± 26%, 65% ± 4%, and 47% ± 19% of total WSOC in Alashan, Xi’an, Qingdao, and Crete during dust events, which are much higher than during non-dust periods (46% ± 12%, 35%, 51% ± 8%, and 25% ± 11%), respectively. Overall, supermicron mode WSOC contributes to an average of 56% ± 18% (range 19%–85%, median 61%) to total WSOC during dust events and 27% ± 13% (range 6%–60%, median 25%) during non-dust periods. Similarly, oxalate was predominantly in supermicron mode during dust events in all locations (Fig. 1m–p).

**Figure 2.**
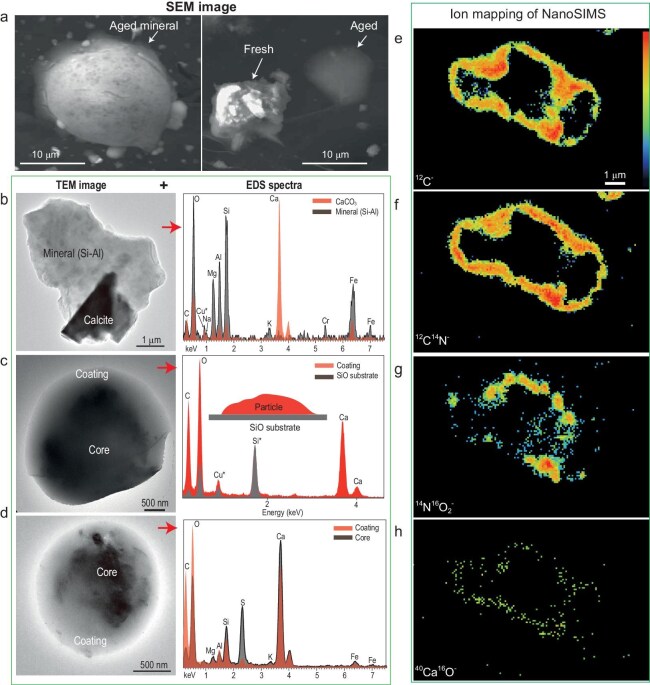
Microscopic observations of aged and fresh mineral dust particles. (a) SEM images showing an aged and a fresh dust particle. (b–d) TEM images and EDS of fresh and aged dust particles: (b) shows a fresh dust particle containing calcite and aluminosilicate minerals, and (c) and (d) an aged dust particle containing aluminosilicate core and Ca-rich coating. Note Cu and Si in the EDS spectra are from copper grid or SiO film substrates in (c) and (d). (e–h) are nanoSIMS maps of ^12^C^−,12^C^14^N^−, 14^N^16^O_2_^−^ and ^40^Ca^16^O^−^, respectively, of an aged dust particle collected at Alashan, confirming the presence of organic matter and nitrate in the coatings.

To better understand why WSOC predominantly exists in the supermicron particles during dust events, we carried out microscopic analyses of individual particles. Scanning electron microscope (SEM) analysis showed that some aged dust particles exhibited a core-shell shape and smooth surface compared to fresh dust particles (Fig. 2a and Fig. S2a). In this study, 1063 dust particles were analyzed by high-resolution transmission electron microscopy (TEM). Figure 2b–d illustrates the most common types of dust particles distinguished by their characteristic morphology and composition: fresh dust particles with irregular shape (Fig. 2b) and aged dust particles with core-shell shape (Fig. 2c, d). In this case, they showed a typical core-shell structure. Energy-dispersive X-ray spectrometry (EDS) indicated that the coating contained C, O, and Ca (Fig. 2c), different from the mineral core which consists of Si, Al, and O (Fig. 2d). In contrast, the fresh dust particles typically do not contain secondary species such as S and N. Figure S2b shows aged dust particles with visible reacted sites and a different surface structure to fresh dust particles (e.g. Fig. 2b). Our results showed that about a third of the dust particles exhibited evidence of ageing during non-dust periods or after dust events, but no direct evidence was observed for dust collected during dust events in Alashan; in contrast, a significant fraction (27%) of supermicron dust particles at the downwind Qingdao site showed evidence of ageing (Table S2 and Fig. S2). During dust events, secondary particles such as sulfate and nitrate were present in submicron fractions (diameter <1 μm), whereas the supermicron fractions predominantly contained dust particles (Figs S3 and S4).

The SEM image showed that the Ca-rich coating likely encapsulated the mineral core, forming the typical core-shell structure (Fig. 2a). Atomic force microscopy (AFM) analysis further confirmed our observations that the core-shell particles display a smooth surface (Fig. S2). Furthermore, nanoscale secondary ion mass spectrometry (NanoSIMS) showed that aged dust particles contained fragments such as C^−^, CN^−^, O^−^, NO_2_^−^, and CaO^−^ in the coating (Fig. 2e–h and Fig. S5), while their core mainly contained O^−^ and Si^−^ fragments (Fig. S5a). No CN^−^ and NO_2_^−^ signals were detected in fresh dust particles (Fig. S6).

### Modelling aqSOA formation on supermicron aerosols

**Figure 3.**
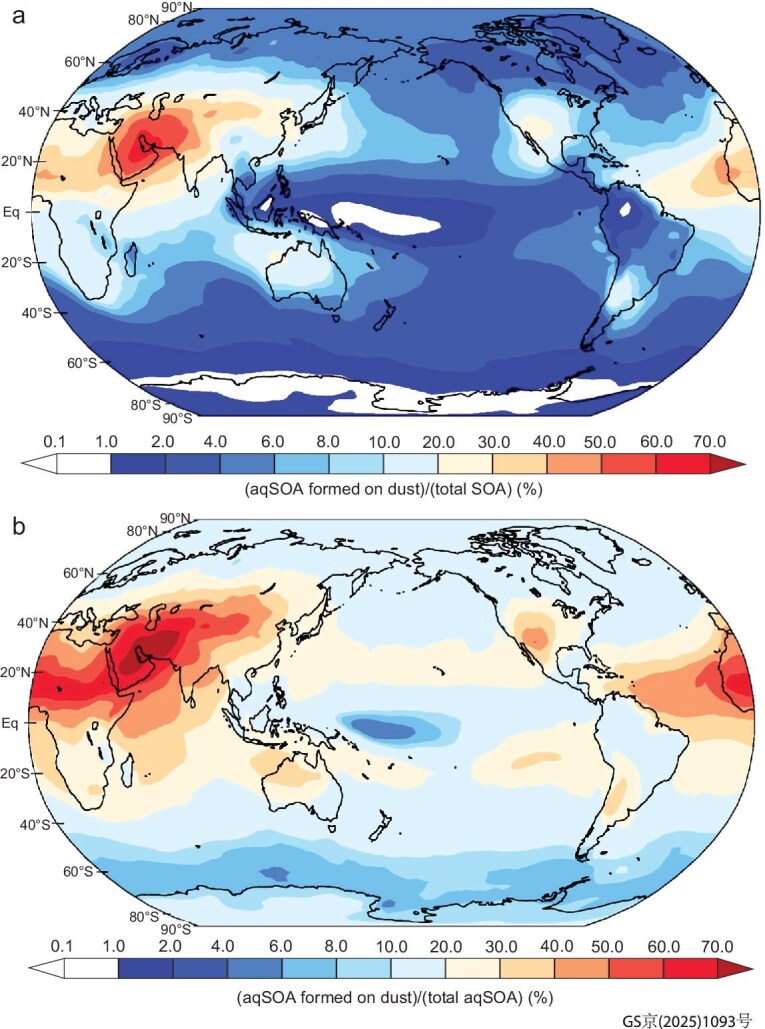
Contribution of global ground-level aqSOA formed on dust to total SOA (a) and total aqSOA (b) simulated by the improved IMPACT model. The contributions of aqSOA formed on dust are higher across the ‘dust belt’, from East Asia to the Middle East and North Africa.

We employed the Integrated Massively Parallel Atmospheric Chemical Transport (IMPACT) model to simulate the mass concentrations of calcite, nitrate, aerosol water, and aqSOA formed on dust (see Methods). The model predictions of aqSOA formation in a multiphase reaction process scheme and a reactive uptake of epoxide, glyoxal, and methylglyoxal on sulfate aerosol have been analyzed in a previous study [17]. When only the condensation and evaporation of water-soluble gases (e.g. glyoxal, methylglyoxal, and glycolaldehyde) on dust is represented in the base model, the aqSOA concentration in supermicron dust is negligible (Fig. 3a, Fig. S7a vs S7b), in contrast to observations (Fig. 1 and Fig. 2). To reproduce observed WSOC fractions in supermicron aerosols, a resistor model is applied in the model to calculate the reactive uptake coefficients of glyoxal and methylglyoxal in the formation of aqSOA on dust (hereafter referred to as the improved model, Fig. S7c and S7d). In contrast to the base model with negligible supermicron model WSOC (coefficient of determination (*R*^2^ = 0.01)), the contributions of supermicron to total WSOC predicted in the improved model (*R*^2^ = 0.84) agreed well with observations in Crete (Fig. S8) (see Methods and SI). The salting-in effect increases the solubility of glyoxal in solution, while the salting-out effect decreases that of methylglyoxal. To consider these effects on the reactive uptake coefficients, sensitivity simulations (*R*^2^ = 0.78) prescribed the measured Henry's law constants for glyoxal and methylglyoxal (see Methods and SI) and overestimated the contribution of supermicron mode to total WSOC. The improved model, which represented aqSOA formation on dust, predicted a 13% higher annual average WSOC mass concentration (0.79 ± 0.69 μg m⁻³) compared to the simulation that excluded dust (0.70 ± 0.60 μg m⁻³). This is consistent with the observations in Crete, which showed that WSOC concentrations were enhanced during the dust days compared to non-dust days by 15% on average (Fig. S9). However, the base simulations indicated similar annual average WSOC mass loadings when either including dust or excluding dust (0.80 ± 0.66 μg m⁻³).

To further evaluate the model performance, we compared the submicron organic aerosol, SOA, oxygen-to-carbon (O/C) ratios in the base and improved model simulations with the observed data at two continental background stations, NamCo and Waliguan [36] (Fig. S10). Overall, the base model significantly overestimated OA and SOA concentrations as well as O/C ratios. Both the improved model and its sensitivity simulation, which dynamically estimated the reactive uptake coefficients of glyoxal and methylglyoxal, predicted lower concentrations of submicron SOA than that from the base model. Consequently, when the base model significantly overestimated the total SOA concentrations, the improved model showed a better agreement (Fig. S10). On the other hand, when the base model reproduced the observed high SOA concentrations well, the improved model showed an underestimate.

Figure 3 illustrates the contribution of aqSOA formed in dust to the total SOA from the simulations. The contribution of aqSOA in supermicron dust to total SOA in the base model is negligible (Fig. S7a). However, the annual average contribution of aqSOA formed on total dust to the total SOA from the simulations in the improved model is two orders of magnitude higher than that in the base model over the land: 16% vs 0.2% (Fig. 3a). The contribution reaches as high as 67% over the ‘dust belt’, from the western Sahara through the Middle East and Central Asia to East Asia (Fig. 3a). The improved model shows the highest contribution of aqSOA on dust to total SOA in the moderately polluted regions downwind of the major deserts, particularly in East Asia (Fig. 3a). The spatial distribution of aqSOA formed in dust to total aqSOA in the improved model (Fig. 3b) is similar to that of total SOA with a contribution reaching as high as 74% over the ‘dust belt’ and 28% for global land.

## DISCUSSIONS

### aqSOA detected in aged Ca nitrate–containing dust particles

The content of WSOC in PM_10_ at the downwind sites (e.g. Alashan and Qingdao) is 0.8% ± 0.5% and 2.7% ± 1.2%, which cannot be explained by the low WSOC in the fresh dust particles (0.27% ± 0.16%) (i.e. PM_10_ collected from the suspended soils from Asian dust source regions) (Fig. S11). These results show that the WSOC observed at the downwind sites is predominantly formed through gas-to-particle conversion during the long-range transport of dust, rather than from primary emissions. This is further supported by the strong correlation between secondary organic carbon (SOC), estimated from the organic carbon/elemental carbon data following Turpin and Huntzicker [37], and the measured WSOC in the supermicron sizes (*R*^2^ = 0.87, and the slope close to 1) during dust events in Crete (Fig. S12a and c).

The shape and chemical composition of the aged dust particles shown in Fig. 2a, b, and d indicate the presence of Ca and nitrate, consistent with previous studies [21,23,28,29,31]. Such Ca nitrate–containing particles are not observed in fresh dust particles in this study (Fig. 2b) nor in numerous previous studies [21,28,29,31]. The nanoSIMS maps confirmed the presence of Ca nitrate using NO_2_^−^ ions as a tracer (Fig. 2g) and organic species using C^−^ and CN^−^ ions as tracers [38] in the same particles. These findings provide indisputable evidence that these coatings of Ca-containing aged dust particles not only contain nitrate but also organic species. In contrast, no organic species were detected in fresh dust particles by TEM-EDS and NanoSIMS analyses (Fig. 2b and Fig. S6). Therefore, we conclude that aqSOA is formed on aged Ca nitrate–containing dust particles during long-range transport.

### Formation mechanisms of aqSOA in aged dust particles

There are several processes that could potentially explain our observations.

1. **Condensation of oxidized organics alone**: Water-soluble organic compounds can condense on all aerosol surfaces including dust particles. However, if the condensation process alone (without further chemical processing) is key to the formation of WSOC, then we would expect them to be uniformly distributed in all particles. This is not the case—we only detected organic matter on aged Ca nitrate–containing particles (Fig. 2). Furthermore, while water-soluble organic gases, such as glyoxal and methylglyoxal, condense on all particles, they also evaporate from the particles. This reversible uptake is often observed for ‘dry’ particles at low relative humidity (RH) [39–41], including those containing typical calcium sulfate or organic salts (such as oxalate) which have a high deliquesce RH (>90%, a RH that is not typically seen during dust events) (Fig. S1 and Table S1) [40]. Therefore, although we cannot rule out the contribution of condensation organic species alone (i.e. without further chemical processing) to the observed WSOC on dust particles, it is unlikely to be the key process for supermicron mode aqSOA formation. This is further supported by the lack of correlation between WSOC and surface area in Crete (Fig. S13) as well as by the negligible WSOC formation in the supermicron mode in the base model, which had already considered the condensation process (Fig. S7a).
2. **Coagulation of SOA with dust**: We observed fine-mode SOA particles from individual particle analysis (Fig. S3). In theory, coagulation of fine-mode aqSOA with dust particles can shift the size distribution towards coarse mode. However, we did not observe organic particles on dust particles at downwind sites under NanoSIMS and TEM (Figs S2 and S6), suggesting that coagulation of SOA with dust is unlikely to be important. This can be explained by the relatively low number of concentrations of dust (even though mass concentration can be high) during dust events [42].
3. **Cloud processing**: Cloud processing has been suggested to be a key process in aqSOA formation in models, mainly in the fine mode [8]. If dust particles are processed in the clouds, aqSOA may form on their surfaces. Here, we found no evidence of cloud processes of particles during the studied dust events: satellite images (Fig. S14) indicate that dust particles during our observed events do not interact with clouds, and the RH in the dust-laden air masses is typically <80% (Fig. S1). These results suggest that cloud processing cannot explain our observed high contribution of coarse-mode WSOC during dust events (Fig. 1i–l).
4. **Reactions on particle surfaces involving absorbed water**: Natural mineral dust is often covered with a monolayer of adsorbed water [43]. It has been hypothesized that this water may be involved in a chemical reaction, leading to formation of secondary species [44]. However, the mass of water adsorbed [45] is typically several times less than aerosol water associated with Ca(NO_3_)_2_ under typical ambient conditions (e.g. RH 40% and 60%) (Tables S1 and S7). Moreover, the uptake coefficient for glyoxal on ‘dry’ dust particles is 2 orders of magnitude lower than those determined on aqueous particles [40]. These results suggest that adsorbed water, even if it is involved in the formation of SOA on ‘dry’ dust particles, is expected to have a limited impact on total SOA formation under ambient RH conditions.
5. **Multiphase reactions**: The fact that organic signals were only detected in the Ca nitrate coatings of aged dust particles suggests an important role of Ca nitrate in SOA formation (Fig. 2). Ca(NO_3_)_2_ is a very special compound, which deliquesces at a RH as low as 8% [31,32,46]. This means that as soon as Ca nitrate is formed on a particle, it starts to absorb water, considering that ambient RH is always above 8% during our field campaigns (Fig. S1); and the water content increases with RH. This is in contrast to major organic acids and their ammoniated or Ca salts, such as Ca oxalate and Ca acetate, with deliquesce RH typically over 90% (see Table S1). The deliquesce RH of the Ca sulfate is also >95% [47]. Thus, none of these species, if formed, will become deliquesced under typical RH during observed dust events. Indeed, aerosol liquid water in the supermicron particles was dominated by water associated with Ca(NO_3_)_2_ during dust events (Fig. S15 and Tables S3–6). Furthermore, Fig. S16 shows that aerosol liquid water (ALW) and WSOC had moderate to high correlations (*r* = 0.62–0.99) during dust days in Alashan, Xi'an, Qingdao, and Crete. However, no correlation was found during non-dust days. This further supported our argument that the aqSOA is formed via aqueous phase processes.

The presence of water in Ca nitrate–containing dust particles enabled the uptake of water-soluble organic compounds through condensation and diffusion. This is illustrated by the detection of glyoxal and methylglyoxal as well as their oxidation product, oxalate, in the particle phase during dust events, which all peaked at the supermicron mode (Fig. S17). Once in aerosol water, water-soluble organic compounds can undergo further chemical processing [31,32,46], including by photochemically generated OH radicals from particulate nitrate photolysis at the air-water interface, leading to the formation of less volatile species, such as organic acids [40,48–50]. However, without further processing, at least some of these compounds, such as formic acid [50], may be volatilized again. In aged carbonate-containing dust particles, this volatilization is suppressed because these acids [8,13,14] form salts after reacting with carbonate. The good correlations between Ca^2+^ and NO_3_^−^ (*R*^2^ = 0.32–0.92) and Ca^2+^ and oxalate (*R*^2^ = 0.46–0.95) in the supermicron particles (Fig. S18) supported this mechanism. Moreover, formate concentrations significantly increased following Ca^2+^ and NO_3_^−^ during the dust periods though at undetectable levels in resuspended desert soil (Fig. S11). Zhang *et al.* [50] demonstrated that particulate nitrate photolysis can produce oxidants (i.e. OH, NO_2_, and NO_2_^−^/HNO_2_) in aqueous droplets, which oxidize glyoxal in a simulated solution to form formic acid/formate. Carlton *et al.* [51] also found that oxalate is another major oxidation product from glyoxal oxidation in aqueous reactions. Furthermore, high solute concentrations in aerosol water create additional chemical pathways (oligomerization) [8,10,11,13] that facilitate the irreversible uptake of organics in the particle phase.

Finally, the detection of organosulfates (OSs), which are considered to be tracers for aqueous reactions [52,53], in supermicron dust, further supported aqueous formation mechanisms of aqSOA. Indeed, the concentrations of total isoprene organosulfates (OSs), monoterpene OSs, and aliphatic OSs, are even higher in supermicron than submicron particles during the two dust events in Qingdao (Fig. S19), indicating a significant production of these OSs on supermicron dust particles. Together, our observations provide strong evidence on the importance of aqueous formation of organic aerosols in the supermicron mode.

Our modelling results also supported the multiphase reaction process mechanisms in wet aerosols. In the base model which considers reversible uptake of glyoxal at the interface between gas and liquid phases and the bulk-phase reactions, the calculated contribution of aqSOA in supermicron aerosols in Crete is only 0.3% ± 0.1% (Fig. S8). This is because the base model accounts for the reactive uptake of glyoxal on sulfate aerosols in the sub-micron mode only. In the improved model with reactive uptake processes on dust, the contributions of aqSOA formed on supermicron aerosols to total SOA (25% ± 4%) agree well with observations in Crete (Fig. S8). The reactive uptake parameters for glyoxal used in the improved model represent the salting effects, aerosol thermodynamics, mass transfer, and aqueous-phase chemical reactions with OH radicals. This implicitly accounts for any chemical reactions of carboxylic acids involving the formation of carboxylate salts and oligomers within the aerosol. Thus, the improved model demonstrates enhanced concentration of SOA in the aged dust over the polluted regions, compared to near the source regions where the fresh dust is emitted with no aqSOA and then is mixed with long-range transported dust (Fig. S7). This explains why a significant portion of aqSOA is formed on aged supermicron dust over the ‘dust belt’ such as the Middle East (Fig. 3).

### Atmospheric implications

**Figure 4.**
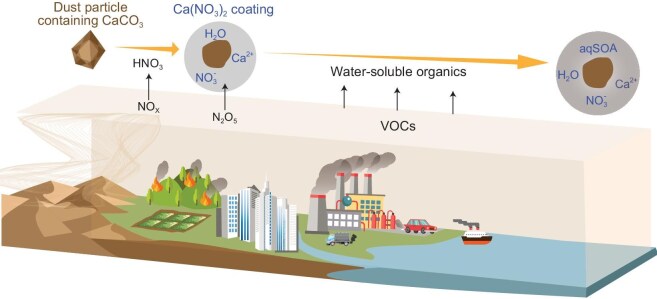
A schematic diagram showing the formation of aqSOA on dust particles. Once in the atmosphere, dust particles containing calcite (i.e. CaCO_3_) react with HNO_3_, formed from primary NO_x_ emissions. This process produces Ca(NO_3_)_2_, which deliquesces at RH as low as 8%. During the night, N_2_O_5_ may also partition to aerosol water which further contributes to HNO_3_ and Ca nitrate formation. Thus, aged Ca(NO_3_)_2_-containing dust particles contain water under normal atmospheric conditions. Water-soluble organics (such as glyoxal) then partitions into the water around such aged dust particles, which is further transformed to aqueous secondary organic aerosol (aqSOA). The aqSOA formation could involve multiple pathways including oxidation, oligomerization, and/or alkaline-acid reactions.

Figure 4 presents a conceptual model illustrating the transport and ageing process of dust particles and the formation of aqSOA. In the atmosphere, dust particles undergo atmospheric ageing, and those containing Ca carbonate react with acids such as HNO_3_ to form Ca(NO_3_)_2_. Then, aged dust particles with Ca(NO_3_)_2_ absorb water under atmospheric conditions (RH >8%), providing a medium for the uptake and oxidation of gaseous water-soluble organics, ultimately resulting in the formation of aqSOA.

The formation of aqSOA on dust enhances SOA formation and shifts the distribution of SOA from submicron to supermicron particles (Fig. 1), with implications on SOA lifetime and thus the burden and spatial distribution of SOA compared to previous models (Fig. 3 and Fig. S7). The uptake of VOCs onto dust can also change atmospheric composition, potentially impacting the ozone budget [24], photochemical reactions of NO_x_ [23,28,29,49], and the formation of submicron SOA and nitrate particles [34,49,54,55]. Furthermore, given that the atmospheric ageing of dust particles may contribute to brown carbon formation [10,11], the reactions on aged mineral dust may also affect aerosol radiative forcing.

The formation of aqSOA also has complex implications for the physical and chemical properties of dust particles. First, aqSOA can influence the properties, such as hygroscopicity, of the dust particles [22,56]. This has the potential to change their ice nucleation ability and CCN activity [4,57,58]. Second, the uptake of VOCs and NOx onto supermicron dust particles might reduce number concentrations or size of sub-micrometer particles, which could reduce CCN concentrations with a potential impact on cloud albedo [5,59]. Third, the ageing process of mineral dust can change a typical irregular shape into more round shape, affecting their optical scattering properties [26]. Fourth, if the aged dust particles contain Fe minerals, the aqSOA (e.g. oxalate) formed on their surfaces can enhance Fe dissolution, thereby delivering more bioavailable Fe to the surface ocean [25]. This may also have implications for health effects of SOA, considering their potential for solubilizing metals and the shift to large sizes.

In summary, we demonstrated the importance of the aqSOA formation process in supermicron dust over the ‘dust belt’ and beyond. The improved model and its sensitivity simulation revealed the significant implications of predicting salting behavior in aqSOA formation processes as those typically contain a mixture of salts. This new process should be incorporated into chemical transport models and their implications are quantified. Further field and laboratory observations are needed to understand the dominant pathways and determine reaction kinetics of aqSOA formation in aged dust, considering the effects of the different salts, and determine the impacts of aqSOA formation on the physical and chemical properties, CCN, ice nucleating ability, and iron availability of dust particles. Such new process understandings should then be developed into models to improve the simulation of the uptake of organic compounds, subsequent aqSOA formation, partitioning of SOA in super- vs sub-micrometer particles, and wider impacts on air quality, marine biogeochemistry, and radiative forcing.

## MATERIALS AND METHODS

### Sample collection and preparation

Individual aerosol particles were collected at a background site near the desert border in Alashan in Inner Mongolia and a polluted site in Qingdao. Alashan in Inner Mongolia (38^o^47’ N, 105^o^21’ E, elevation: 1299 m), which is close to the Tengger desert border (Fig. S1), was chosen to collect aerosol particles in dust and non-dust days during 16–17 April, 2015; 18–23 April, 2015; 20–30 June, 2019; and 2–16 April, 2021. The size-resolved bulk samples were collected in Alashan, Xi'an, Qingdao, and Crete (Greece). Nine sets of size-resolved aerosol samples were collected in Alashan: 3 sets during 20–30 June, 2019, and 6 sets during 2–16 April, 2021. Four sets of samples were collected during dust events (Fig. 1).

### Offline microscopic and spectroscopic analysis

1. *Scanning electron microscopy (SEM).* Individual particle samples were analyzed using a Zeiss Ultra 55 SEM with a field emission gun operating at 5–20 kV. The SEM was equipped with an energy-dispersive X-ray spectrometry (EDS) which can check the chemical composition of individual particles. In this study, the SEM was used to obtain information on the morphology of individual aged dust particles [21,47] (Fig. 2).
2. *Transmission electron microscopy (TEM).* Individual particle samples were examined by a JEOL JEM-2100 transmission electron microscope operated at 200 kV with an energy-dispersive X-ray spectrometry (TEM/EDS).
3. *Nanoscale secondary ion mass spectrometry (NanoSIMS).* Aerosol samples were additionally analysed using a CAMECA NanoSIMS 50 L. A beam of ^133^Cs^+^ primary ions with current of 1–2 pA and beam diameter of ∼100 nm was utilized during the measurements.

### Bulk chemical analysis and aerosol water simulation

For the size-resolved samples collected at the Alashan site, an ion chromatography system (Dionex ICs-600, USA) and a Shimadzu TOC-L analyzer were used to determine the mass concentrations of water-soluble ions (i.e. Ca^2+^, Mg^2+^, K^+^, Na^+^, NH_4_^+^, NO_3_^−^, SO_4_^2−^, Cl^−^, and F^−^) and water-soluble organic carbon (WSOC), respectively.

The ISORROPIA II model (https://www.epfl.ch/labs/lapi/models-and-software/isorropia/) was used to simulate aerosol liquid water (ALW) in size-resolved aerosols based on the SO_4_^2−^-NO_3_^−^-NH_4_^+^-H_2_O inorganic system. The inputs include meteorological factors (temperature and relative humidity) and chemical species (i.e. SO_4_^2−^, NO_3_^−^, Cl^−^, Na^+^, NH_4_^+^, and Ca^2+^). ALW can be derived directly from the outputs.

### Atmospheric modelling

This study used the Integrated Massively Parallel Atmospheric Chemical Transport (IMPACT) model. The model simulations were performed using a horizontal resolution of 2.0° × 2.5° for latitude by longitude and 47 vertical layers. The chemical transport model was driven by the Modern Era Retrospective analysis for Research and Applications 2 (MERRA-2) reanalysis of meteorological data from the National Aeronautics and Space Administration (NASA) Global Modeling and Assimilation Office (GMAO). The detailed setup is shown in Supplementary materials.

The multiphase reaction process scheme is used to predict the net aqSOA production in aerosol and cloud water, whereas the irreversible uptake of epoxide, glyoxal, and methylglyoxal is considered on sulfate aerosol only in the base simulation. To estimate the reactive uptake of glyoxal and methylglyoxal on the five externally mixed aerosols, a resistor model is used in the improved simulation. To examine the sensitivity of reactive uptake coefficients for glyoxal (and methylglyoxal) to the salting-in (salting-out) effects, the measured Henry's law constants are prescribed for the resistor model in the sensitivity simulation.

## Supplementary Material

nwaf221_Supplemental_File

## References

[1] Jimenez JL, Canagaratna MR, Donahue NM et al. Evolution of organic aerosols in the atmosphere. Science 2009; 326: 1525–9. 10.1126/science.1180353 20007897

[2] Huang R-J, Zhang Y, Bozzetti C et al. High secondary aerosol contribution to particulate pollution during haze events in China. Nature 2014; 514: 218–22. 10.1038/nature13774 25231863

[3] Lamkaddam H, Dommen J, Ranjithkumar A et al. Large contribution to secondary organic aerosol from isoprene cloud chemistry. Sci Adv 2021; 7: eabe2952. 10.1126/sciadv.abe2952 33762335 PMC7990335

[4] Kanakidou M, Seinfeld JH, Pandis SN et al. Organic aerosol and global climate modelling: a review. Atmos Chem Phys 2005; 5: 1053–123. 10.5194/acp-5-1053-2005

[5] Zhu J, Penner JE, Lin G et al. Mechanism of SOA formation determines magnitude of radiative effects. Proc Natl Acad Sci USA 2017; 114: 12685–90. 10.1073/pnas.1712273114 29133426 PMC5715767

[6] Nie W, Yan C, Huang DD et al. Secondary organic aerosol formed by condensing anthropogenic vapours over China's megacities. Nat Geosci 2022; 15: 255–61. 10.1038/s41561-022-00922-5

[7] Kelly JM, Doherty RM, O'Connor FM et al. The roles of volatile organic compound deposition and oxidation mechanisms in determining secondary organic aerosol production: a global perspective using the UKCA chemistry–climate model (vn8.4). Geosci Model Dev 2019; 12: 2539–69. 10.5194/gmd-12-2539-2019

[8] Ervens B, Turpin BJ, Weber RJ. Secondary organic aerosol formation in cloud droplets and aqueous particles (aqSOA): a review of laboratory, field and model studies. Atmos Chem Phys 2011; 11: 11069–102. 10.5194/acp-11-11069-2011

[9] Franco B, Blumenstock T, Cho C et al. Ubiquitous atmospheric production of organic acids mediated by cloud droplets. Nature 2021; 593: 233–7. 10.1038/s41586-021-03462-x 33981052 PMC8116209

[10] Herrmann H, Schaefer T, Tilgner A et al. Tropospheric aqueous-phase chemistry: kinetics, mechanisms, and its coupling to a changing gas phase. Chem Rev 2015; 115: 4259–334. 10.1021/cr500447k 25950643

[11] Zhang R, Gen M, Liang Z et al. Photochemical reactions of glyoxal during particulate ammonium nitrate photolysis: brown carbon formation, enhanced glyoxal decay, and organic phase formation. Environ Sci Technol 2022; 56: 1605–14. 10.1021/acs.est.1c07211 35023733

[12] Gilardoni S, Massoli P, Paglione M et al. Direct observation of aqueous secondary organic aerosol from biomass-burning emissions. Proc Natl Acad Sci USA 2016; 113: 10013–8. 10.1073/pnas.1602212113 27551086 PMC5018753

[13] Lim YB, Tan Y, Perri MJ et al. Aqueous chemistry and its role in secondary organic aerosol (SOA) formation. Atmos Chem Phys 2010; 10: 10521–39. 10.5194/acp-10-10521-2010

[14] Xu B, Zhang G, Gustafsson Ö et al. Large contribution of fossil-derived components to aqueous secondary organic aerosols in China. Nat Commun 2022; 13: 5115. 10.1038/s41467-022-32863-3 36045131 PMC9433442

[15] Hodzic A, Campuzano-Jost P, Bian H et al. Characterization of organic aerosol across the global remote troposphere: a comparison of ATom measurements and global chemistry models. Atmos Chem Phys 2020; 20: 4607–35. 10.5194/acp-20-4607-2020

[16] Lin G, Sillman S, Penner JE et al. Global modeling of SOA: the use of different mechanisms for aqueous-phase formation. Atmos Chem Phys 2014; 14: 5451–75. 10.5194/acp-14-5451-2014

[17] Marais EA, Jacob DJ, Jimenez JL et al. Aqueous-phase mechanism for secondary organic aerosol formation from isoprene: application to the Southeast United States and co-benefit of SO_2_ emission controls. Atmos Chem Phys 2016; 16: 1603–18. 10.5194/acp-16-1603-2016 32742280 PMC7394309

[18] Ervens B, Volkamer R. Glyoxal processing by aerosol multiphase chemistry: towards a kinetic modeling framework of secondary organic aerosol formation in aqueous particles. Atmos Chem Phys 2010; 10: 8219–44. 10.5194/acp-10-8219-2010

[19] Myriokefalitakis S, Tsigaridis K, Mihalopoulos N et al. In-cloud oxalate formation in the global troposphere: a 3-D modeling study. Atmos Chem Phys 2011; 11: 5761–82. 10.5194/acp-11-5761-2011

[20] Fu T-M, Jacob DJ, Wittrock F et al. Global budgets of atmospheric glyoxal and methylglyoxal, and implications for formation of secondary organic aerosols. J Geophys Res 2008; 113: D15303.

[21] Tobo Y, Zhang D, Matsuki A et al. Asian dust particles converted into aqueous droplets under remote marine atmospheric conditions. Proc Natl Acad Sci USA 2010; 107: 17905–10. 10.1073/pnas.1008235107 20921372 PMC2964202

[22] Sullivan RC, Moore MJK, Petters MD et al. Effect of chemical mixing state on the hygroscopicity and cloud nucleation properties of calcium mineral dust particles. Atmos Chem Phys 2009; 9: 3303–16. 10.5194/acp-9-3303-2009

[23] Laskin A, Wietsma TW, Krueger BJ et al. Heterogeneous chemistry of individual mineral dust particles with nitric acid: a combined CCSEM/EDX, ESEM, and ICP-MS study. J Geophys Res 2005; 110: 10208.

[24] Usher CR, Michel AE, Grassian VH. Reactions on mineral dust. Chem Rev 2003; 103: 4883–940. 10.1021/cr020657y 14664636

[25] Myriokefalitakis S, Bergas-Massó E, Gonçalves-Ageitos M et al. Multiphase processes in the EC-Earth model and their relevance to the atmospheric oxalate, sulfate, and iron cycles. Geosci Model Dev 2022; 15: 3079–120. 10.5194/gmd-15-3079-2022

[26] Kok JF, Storelvmo T, Karydis VA et al. Mineral dust aerosol impacts on global climate and climate change. Nat Rev Earth Environ 2023; 4: 71–86. 10.1038/s43017-022-00379-5

[27] Lin G, Penner JE, Zhou C. How will SOA change in the future? Geophys Res Lett 2016; 43: 1718–26. 10.1002/2015GL067137

[28] Li WJ, Shao LY. Observation of nitrate coatings on atmospheric mineral dust particles. Atmos Chem Phys 2009; 9: 1863–71. 10.5194/acp-9-1863-2009

[29] Krueger BJ, Grassian VH, Cowin JP et al. Heterogeneous chemistry of individual mineral dust particles from different dust source regions: the importance of particle mineralogy. Atmos Environ 2004; 38: 6253–61. 10.1016/j.atmosenv.2004.07.010

[30] Falkovich AH, Schkolnik G, Ganor E et al. Adsorption of organic compounds pertinent to urban environments onto mineral dust particles. J Geophys Res 2004; 109: D02208. 10.1029/2003JD003919

[31] Sullivan RC, Prather KA. Investigations of the diurnal cycle and mixing state of oxalic acid in individual particles in Asian aerosol outflow. Environ Sci Technol 2007; 41: 8062–9. 10.1021/es071134g 18186338

[32] Wang G, Cheng C, Meng J et al. Field observation on secondary organic aerosols during Asian dust storm periods: formation mechanism of oxalic acid and related compounds on dust surface. Atmos Environ 2015; 113: 169–76. 10.1016/j.atmosenv.2015.05.013

[33] Xu W, Kuang Y, Liang L et al. Dust-dominated coarse particles as a medium for rapid secondary organic and inorganic aerosol formation in highly polluted air. Environ Sci Technol 2020; 54: 15710–21. 10.1021/acs.est.0c07243 33237756

[34] Deshmukh DK, Kawamura K, Kobayashi M et al. Changes in the size distributions of oxalic acid and related polar compounds over Northern Japan during spring. J Geophys Res Atmospheres 2023; 128: e2022JD038461. 10.1029/2022JD038461

[35] Li H, Zhang Q, Jiang W et al. Characteristics and sources of water-soluble organic aerosol in a heavily polluted environment in Northern China. Sci Total Environ 2021; 758: 143970. 10.1016/j.scitotenv.2020.143970 33338790

[36] Xu J, Zhang X, Zhao W et al. High-resolution physicochemical dataset of atmospheric aerosols over the Tibetan Plateau and its surroundings. Earth Syst Sci Data 2024; 16: 1875–900. 10.5194/essd-16-1875-2024

[37] Turpin BJ, Huntzicker JJ. Identification of secondary organic aerosol episodes and quantitation of primary and secondary organic aerosol concentrations during SCAQS. Atmos Environ 1995; 29: 3527–44. 10.1016/1352-2310(94)00276-Q

[38] Pöhlker C, Wiedemann KT, Sinha B et al. Biogenic potassium salt particles as seeds for secondary organic aerosol in the Amazon. Science 2012; 337: 1075–8. 10.1126/science.1223264 22936773

[39] Nah T, McVay RC, Zhang X et al. Influence of seed aerosol surface area and oxidation rate on vapor wall deposition and SOA mass yields: a case study with pinene ozonolysis. Atmos Chem Phys 2016; 16: 9361–79. 10.5194/acp-16-9361-2016

[40] Zogka AG, Lostier A, Papadimitriou VC et al. Unraveling the uptake of glyoxal on a diversity of natural dusts and surrogates: linking dust composition to glyoxal uptake and estimation of atmospheric lifetimes. ACS Earth Space Chem 2024; 8: 1165–78. 10.1021/acsearthspacechem.3c00359

[41] Battaglia F, Formenti P, Giorio C et al. Formation and composition of organic aerosols from the uptake of glyoxal on natural mineral dust aerosols: a laboratory study. EGUsphere [Preprint], 10.5194/egusphere-2024-4073.

[42] Mahowald N, Albani S, Kok JF et al. The size distribution of desert dust aerosols and its impact on the Earth system. Aeolian Res 2014; 15: 53–71. 10.1016/j.aeolia.2013.09.002

[43] Joshi N, Romanias MN, Riffault V et al. Investigating water adsorption onto natural mineral dust particles: linking DRIFTS experiments and BET theory. Aeolian Res 2017; 27: 35–45. 10.1016/j.aeolia.2017.06.001

[44] Zeineddine MN, Urupina D, Romanias MN et al. Uptake and reactivity of acetic acid on Gobi dust and mineral surrogates: a source of oxygenated volatile organic compounds in the atmosphere? Atmos Environ 2023; 294: 119509. 10.1016/j.atmosenv.2022.119509

[45] Chen L, Peng C, Gu W et al. On mineral dust aerosol hygroscopicity. Atmos Chem Phys 2020; 20: 13611–26. 10.5194/acp-20-13611-2020

[46] Wang B, Laskin A. Reactions between water-soluble organic acids and nitrates in atmospheric aerosols: recycling of nitric acid and formation of organic salts. J Geophys Res 2014; 119: 3335–51.

[47] Shi Z, Zhang D, Hayashi M et al. Influences of sulfate and nitrate on the hygroscopic behaviour of coarse dust particles. Atmos Environ 2008; 42: 822–7. 10.1016/j.atmosenv.2007.10.037

[48] Li K, Guo Y, Nizkorodov A et al. Spontaneous dark formation of OH radicals at the interface of aqueous atmospheric droplets. Proc Natl Acad Sci USA 2023; 120: e2220228120. 10.1073/pnas.2220228120 37011187 PMC10104570

[49] Yu Z, Jang M. Atmospheric processes of aromatic hydrocarbons in the presence of mineral dust particles in an urban environment. ACS Earth Space Chem 2019; 3: 2404–14. 10.1021/acsearthspacechem.9b00195

[50] Zhang R, Gen M, Fu TM et al. Production of formate via oxidation of glyoxal promoted by particulate nitrate photolysis. Environ Sci Technol 2021; 55: 5711–20. 10.1021/acs.est.0c08199 33861585

[51] Carlton AG, Turpin BJ, Altieri KE et al. Atmospheric oxalic acid and SOA production from glyoxal: results of aqueous photooxidation experiments. Atmos Environ 2007; 41: 7588–602. 10.1016/j.atmosenv.2007.05.035

[52] McNeill VF, Woo JL, Kim DD et al. Aqueous-phase secondary organic aerosol and organosulfate formation in atmospheric aerosols: a modeling study. Environ Sci Technol 2012; 46: 8075–81. 10.1021/es3002986 22788757

[53] Wang Y, Zhao Y, Wang Y et al. Organosulfates in atmospheric aerosols in Shanghai, China: seasonal and interannual variability, origin, and formation mechanisms. Atmos Chem Phys 2021; 21: 2959–80. 10.5194/acp-21-2959-2021

[54] Zhai S, Jacob DJ, Pendergrass DC et al. Coarse particulate matter air quality in East Asia: implications for fine particulate nitrate. Atmos Chem Phys 2023; 23: 4271–81. 10.5194/acp-23-4271-2023

[55] Laskina O, Young MA, Kleiber PD et al. Infrared extinction spectroscopy and micro-Raman spectroscopy of select components of mineral dust mixed with organic compounds. J Geophys Res Atmospheres 2013; 118: 6593–606. 10.1002/jgrd.50494

[56] Drozd G, Woo J, Häkkinen SAK et al. Inorganic salts interact with oxalic acid in submicron particles to form material with low hygroscopicity and volatility. Atmos Chem Phys 2014; 14: 5205–15. 10.5194/acp-14-5205-2014

[57] Mohler O, Benz S, Saathoff H et al. The effect of organic coating on the heterogeneous ice nucleation efficiency of mineral dust aerosols. Environ Res Lett 2008; 3: 025007. 10.1088/1748-9326/3/2/025007

[58] Kanji ZA, Florea O, Abbatt JPD. Ice formation via deposition nucleation on mineral dust and organics: dependence of onset relative humidity on total particulate surface area. Environ Res Lett 2008; 3: 025004. 10.1088/1748-9326/3/2/025004

[59] Maria SF, Russell LM, Gilles MK et al. Organic aerosol growth mechanisms and their climate-forcing implications. Science 2004; 306: 1921–4. 10.1126/science.1103491 15591199

