# Supplementary material for: Aqueous-phase secondary organic aerosol formation on mineral dust
Source: Natl Sci Rev. 2025 May 31;12(7):nwaf221. doi: 10.1093/nsr/nwaf221 (PMC12258144; doi:10.1093/nsr/nwaf221)
Supplement: nwaf221_Supplemental_File [file nwaf221_supplemental_file.pdf]

Supplementary materials

Aqueous-phase secondary organic aerosol formation on mineral dust

Weijun Li[1*], Akinori Ito[2*], Guochen Wang[1], Minkang Zhi[1], Liang Xu[1], Qi Yuan[1], Jian Zhang[1], Lei Liu[1], Feng Wu[3], Alexander Laskin[4], Daizhou Zhang[5], Xiaoye Zhang[6], Tong Zhu[7], Jianmin Chen[8], Nikolaos Mihalopoulos[9,10], Aikaterini Bougiatioti[9,10], Maria Kanakidou[10,11,12], Gehui Wang[13], Huilin Hu[14], Yue Zhao[14], Zongbo Shi[15,16*]

[1]Department of Atmospheric Sciences, School of Earth Sciences and State Key Laboratory of Ocean Sensing, Zhejiang University, Hangzhou, Zhejiang 310027, China
2Yokohama Institute for Earth Sciences, JAMSTEC, Yokohama, Kanagawa, 236-0001, Japan
[3]Key Laboratory of Aerosol Chemistry & Physics and State Key Laboratory of Loess and Quaternary Geology, Institute of Earth Environment, Chinese Academy of Science, Xi'an, China
[4]Department of Chemistry, Purdue University, West Lafayette, IN 47907, USA.
[5]Faculty of Environmental and Symbiotic Sciences, Prefectural University of Kumamoto, Kumamoto 862-8502, Japan
[6]Key Laboratory of Atmospheric Chemistry of CMA, Institute of Atmospheric Composition, Chinese Academy of Meteorological Sciences, Beijing 100081, China
[7]College of Environmental Sciences and Engineering, Peking University, Beijing, 100871, China
[8]Shanghai Key Laboratory of Atmospheric Particle Pollution and Prevention, Department of Environmental Science and Engineering, Fudan University, Shanghai 200433, China
[9]Institute for Environmental Research and Sustainable Development, National Observatory of Athens, Athens, Greece
[10]Environmental Chemical Processes Laboratory, Department of Chemistry, University of Crete, Greece
[11]Institute of Environmental Physics, Department of Physics, University of Bremen, Germany
[12]Center for the Study of Air Quality and Climate Change (C-STACC), Institute of Chemical Engineering Sciences (ICE-HT), Foundation for Research and Technology, Hellas) (FORTH), Patras, Greece
[13]Key Lab of Geographic Information Science of the Ministry of Education, School of Geographic Sciences, East China Normal University, Shanghai 210062, China
[14]School of Environmental Science and Engineering, Shanghai Jiao Tong University, Shanghai 200240, China
[15]School of Geography, Earth and Environmental Sciences, University of Birmingham, UK
[16]Anhui Institute of Optics and Fine Mechanics, Chinese Academy of Sciences, 231137, Hefei China

Materials and Methods

Sample collection and preparation

At these sampling sites, aerosol particles were collected onto copper TEM grids coated with carbon film (carbon type B, 300-mesh copper, Tianld Co., China) by a two-stage impactor with the D_{50} cut-off sizes of 0.30 μm and 0.08 μm for the first and the second stages, respectively. We typically collected aerosol samples using the TEM grid coated by SiO film for TEM observation and silicon wafer for SEM observation in Qingdao and Alashan. Upon examining the back trajectories of air masses, it was evident that RHs consistently stayed below 80% throughout all dust events prior to the arrival of air masses from dust regions at the sampling sites (Supplementary Fig. 1).

A MOUDI (Micro-Orifice Uniform Deposit Impactor with rotator, TSI 120R) was used to collect the aerosol particles on quartz filters at a flow rate of 30 L min^{-1}. The MOUDI has 10 stages with cut size at 0.056, 0.1, 0.18, 0.32, 0.56, 1.0, 1.8, 3.2, 5.6, and 10 μm. Before sampling, the quartz filters were pre-heated to 500 °C using a muffle furnace to remove residual organics. After sampling, the samples were placed in plastic Petri dishes and stored in a refrigerator at a temperature below -5 °C for subsequent gravimetric and chemical analysis in the laboratory.

94 sets of size-resolved aerosol samples were collected at the Finokalia research station in Crete from July 2004 until February 2007, in which nine sets of samples were collected during African dust events and 85 sets of samples during non-dust periods (Fig. 1). The size-resolved samples were collected for 2 days in each set at an airflow rate of 11 L min^{-1} with 12 size bins with cutoff diameter of 0.041, 0.085, 0.138, 0.225, 0.346, 0.585, 0.762, 1.06, 1.66, 2.68, 4.08 and 8.39 μm (stages 1-12 respectively) during non-dust periods. The station is considered as a remote marine background site, established and operated by the University of Crete. More detailed description of sampling and analysis is shown in Bougiatioti et al.[1].

Eight sets of size-resolved aerosol samples were collected at an urban site in Xi'an, China, in which three sets of samples were collected during dust events. The dust events have been identified by the Chinese Meteorological Administration and publicly reported as one heavy air pollution on 24 April, 2009 and on 29-30 March, 2011 in Xi'an. The size-resolved samples were collected for 4 days in each set at an airflow rate of 28 L min^{-1} with 9 size bins with a cutoff diameter of 0.4, 0.7, 1.1, 2.1, 3.3, 4.7, 5.8, 9.0 and 10.0 μm during non-dust periods. The sampler was set on the rooftop of a three-story building (about 12 m above ground) in the urban area of Xi'an. The site is the downwind of Asian dust source regions and ~560 km far away from the Tengger desert (Supplementary Fig. 1). The dust samples were collected during two strong Asia dust events in Xi'an on 24 April, 2009 and on 29-30 March, 2011, and other (non-dust) samples were collected from 25 March to 25 April, 2009. More sampling details is given in Wang et al.[2, 3].

Five sets of size-resolved aerosol samples were collected at an urban site in Qingdao, China,

in which three sets of samples were collected during Asian dust events in spring, 2023 and two sets of samples were collected during non-dust storm periods. The MOUDI was deployed to collect the aerosol particles on quartz filters at a flow rate of 30 L min^{-1}. The sampling procedure at the coastal Qingdao site was the same to that in Alashan.

We also collected PM_{10} samples during Asian dust periods in Alashan and Qingdao along the dust transport pathway in March-April, 2021-2023 (Supplementary Fig. 1 and Supplementary Fig. 10). In order to understand whether there were organics in the fresh dust particles, we collected three surface soil samples (0–5 cm) from six desert soil samples from the Tengger Desert. All soil samples were sealed and stored in sampling bags at room temperature after air-drying without screening or chemical pretreatments. A laboratory system was set up to generate dust particles with simulated natural wind erosion processes from surface soils or directly from desert surfaces. One aerosol sampler has been connected into the laboratory system to collect the PM_{10} from the resuspended soil. The detailed procedure of the dust sampling and the laboratory system have been introduced by Wu et al.,[4].

Offline microscopic and spectroscopic analysis

TEM can observe the mixing structure of different aerosol components within an individual particle on the substrate. EDS spectra were acquired within a maximum time of 30 s. TEM grids are made of copper (Cu) and covered by a carbon-reinforced substrate, so Cu is excluded from the quantitative analyses of the particle composition. C signal from individual particles is partially from the carbon film coated on the TEM grid. To confirm the presence of carbon in the coating of aged dust particles, dust particles collected on TEM grid coated by SiO film were also analyzed (Fig. 2C). In this study, 1240 mineral dust particles were analyzed.

The instrument was set to simultaneous secondary ion collection mode with pulse counting on electron multipliers, enabling acquisition of seven secondary ions originating from the sputtered area of the sample. A micro-cesium source was used to generate Cs^+ primary ions, with an impact energy of 16 kV for sample interrogation. $^{12}C^-$, $^{16}O^-$, $^{12}C^{14}N^-$, $^{28}Si^-$, $^{14}N^{16}O_2^-$, $^{32}S^-$, and $^{40}Ca^{16}O^-$ ions in individual particles were detected with electron multipliers in a multi-collection mode. A mass resolution of ~6000 (M/ΔM) was used to remove the potential mass interference. Fig. 2 shows the different ion images of individual particles with lateral resolution of ~100 nm.

In this study, we also prepared standard $Ca(NO_3)_2$ samples on TEM grids and Si-wafer. Aerosol particles were generated from the $Ca(NO_3)_2$ solution (1M) using an atomizer (Badger Airbrushes 150-7) and collected onto substrates (Supplementary Figs. 2C&D). Laboratory generated $Ca(NO_3)_2$ particles on TEM grid analyzed by NanoSIMS indicated that $^{14}N^{16}O_2^-$ and $^{40}Ca^{16}O^-$ ions are relevant markers of these particles (Supplementary Figs. 2C&D). $^{12}C^-$ and $^{12}C^{14}N^-$ are used to represent organics in individual particles[5-8].

Bulk chemical analysis

All filters and solutions were kept frozen during transportation to prevent any loss due to volatilization. Filters were extracted ultrasonically for 30 min with 15 mL ultrapure water in all laboratories and then filtered before IC and WSOC analysis. The extraction details including the purity of ultrapure water, model and power of ultrasonicator, and type of syringe filter, vials used for analysis, the calibration detail and quality assurance and quality control (QA-QC) procedures have been reported by Xu et al.[9].

For the size-resolved samples collected at Crete, several stages were combined to ensure there are sufficient materials for analysis: stages 1-3 (A), stage 4 (B), stage 5 (C), stages 6-8 (D), stages 9-10 (E) and stages 11-12 (F). Samples were extracted in ultrasonic bath using 12 ml of nanopure water and then filtered using syringe filters (PALL IC Acrodisc (PES), 0.45 mm, 13 mm) to remove any non-soluble species and subsequently analyzed by ion chromatography and a carbon analyzer. Calibration detail and quality assurance and control (QA, QC) procedures are given in Bougiatioti et al.[1].

For samples collected at Xi'an, each filter was extracted with Milli-Q pure water three times, each in 15 mins under ultrasonication. Then the combined extracts were determined for inorganic ions using an ion chromatograph (Dionex-600) and WSOC using a Shimadzu TOC/N-5000 analyzer. Calibration detail and quality assurance and control (QA, QC) procedures are given in Wang et al.[2, 3]. Dicarboxylic acids, keto-carboxylic acids and a-dicarbonyls in the size-resolved samples were quantified[2, 3]. Briefly, one fourth of the filter was cut in pieces and extracted with Mill-Q pure water for three times each lasting for 15 min. Then the combined extract were concentrated by drying, followed by a reaction with 14% BF3/butanol at 100 ºC for one hour. Afterward, the derivatives were dissolved in hexane and washed with Mill-Q pure water for three times. The hexane layer was further concentrated and analyzed using a gas chromatograph (GC) equipped with a FID detector. Compounds in the samples were also verified using gas chromatography-mass spectrometry (GC/MS) (Supplementary Fig. 4).

To obtain the major organic species in WSOC, we analyzed the size-resolved samples collected in Alashan and Qingdao and PM_{10} samples collected from the suspended Tengger soils and in Alashan and Qingdao during the Asian dust periods. We obtained formate, acetate, and oxalate in an anion ion chromatography system (Dionex ICs-600, USA). Samples were injected into the ion chromatography system containing an analytical column (IonPac AS22 with 4×250 mm) with a guard column (IonPac AG22 with 4×50 mm). Carbonate solution, 1.2 mM $NaHCO_3$/4.0 mM Na_2CO_3 as eluent[10], was used to determine formate, acetate, and oxalate at a flow rate of 1.0 mL min^{-1} during the analyses.

Half of each filter from MOUDI stage was extracted with methanol in an ultrasonic bath for 30 min. To guarantee enough samples, we combine all the filters from the submicron and supermicron particles to analyze the organosulfates (Supplementary Fig. 15). Organosulfates

were analyzed using an Acquity UPLC (Waters, USA) coupled to a Xevo G2-XS Quadrupole time-of-flight mass spectrometer (ToF-MS, Waters, USA) equipped with an electrospray ionization (ESI) source operated in the negative ion mode. The chromatographic conditions and analytical procedures were detailed in our recent publication[11]. A total of 50 organosulfates were identified by UPLC-MS analysis, in which 47 organosulfates (OS) species were quantified. Recent studies show that the OS species are good tracer compounds for aqueous aerosol-phase chemistry[11, 12]. 2-MT-OS is mainly distributed in submicron particles, and its generation mechanism is relatively clear, primarily originating from the catalytic ring-opening reaction of IEPOX acid. Therefore, it may be difficult to generate in alkaline dust particles (Supplementary Fig. 15). C2/C3 OS is also mainly distributed in submicron particles (Supplementary Fig. 15). It may originate from the heterogeneous reaction between 2-MT-OS and OH radicals, and it can also be generated through liquid-phase chemical processes involving C2/C3 organic compounds and sulfur-containing components. Their enrichment in submicron particles may be due to the mixing of upwind dust particles with background submicron particles rich in C2/C3 OS. In dusty days, the atmospheric oxidizing capacity decreases, leading to a weakening of heterogeneous oxidation by OH radicals and the generation of small molecular OS from C2/C3 organic compounds on dust surfaces. This inhibition may not apply to other categories of OS, which may not require high atmospheric oxidizing conditions or highly aged air masses. The concentrations of Isoprene OS, Monoterpene OS, Aliphatic OS and total OSs are higher in supermicron particles (Supplementary Fig. 15). Supplementary Fig. 15 shows that the concentration ratio of supermicron vs submicron OSs is around 1, suggesting the presence of OS generation on dust particles, possibly through the aqueous-phase process.

Atmospheric modelling

The chemical transport model was driven by the Modern Era Retrospective analysis for Research and Applications 2 (MERRA-2) reanalysis meteorological data from the National Aeronautics and Space Administration (NASA) Global Modeling and Assimilation Office (GMAO)[13]. Five externally mixed aerosols are used for the aerosol chemistry in the smallest size bin (<1.26 μm of diameter): (1) sulfates formed from nucleation processes (including direct sulfate emission); (2) carbonaceous aerosols (i.e., primary organic matters and black carbon) from fossil fuel combustion; (3) carbonaceous aerosols from biomass burning, natural sources, and secondary formation; (4) mineral dust; and (5) sea spray aerosols. Three types are used in the larger size bins (1.26–2.5 μm, 2.5–5 μm, and 5–20 μm of diameter): (1) combustion aerosols, (2) mineral dust, and (3) sea spray aerosols. The inorganic compounds (i.e., nitrate, ammonium, and sulfate) and aqSOA are internally mixed with all aerosol types in each size bin. Dust emissions were dynamically simulated using a physically based emission scheme

with the soil mineralogical map. Atmospheric processing from dust sources was projected for four distinct aerosol size bins. The IMPACT model includes the microphysics of sulfate aerosol and the thermodynamics of ammonium, nitrate, and aerosol water coupled to an integrated numerical solution for gas-phase and aqueous-phase photochemistry of organic compounds[14-18]. Sulfate, nitrate and ammonium are highly hygroscopic, and thus can absorb water to form aqueous solutions on dust under certain atmospheric conditions, whereas less hygroscopic materials of organic acids were not treated in the thermodynamic equilibrium model [19, 20] considering their high deliquescence relative humidity (see Supplementary Table 1)

In the base model, we have four different SOA formation pathways: (i) SOA from the gas-particle partitioning of semi-volatile organic compounds (SVOCs), (ii) aerosol phase reactions of these condensed SVOCs, (iii) the nonreactive uptake of water-soluble gases (e.g., glyoxal, methylglyoxal, and glycolaldehyde) on five externally mixed aerosols, and (iv) the reactive uptake of epoxide, glyoxal and methylglyoxal on sulfate aerosol.

In the presence of cloud droplets, water-soluble gases (e.g., glyoxal, methylglyoxal, and glycolaldehyde) dissolve in the aqueous phase and are further oxidized by OH and NO_3 radicals to form products with lower volatility (e.g., dicarboxylic acids and oligomers)[21, 22]. These low volatility products are assumed to remain entirely in the particulate phase as aqSOA when water is evaporated. The aqSOA mass concentration formed in cloud water is proportionally distributed to the aqSOA mass concentration on the pre-existing mineral dust and other types of aerosols. Six aqSOA components were predicted: glyoxylic acid, pyruvic acid, oxalic acid, and three classes of oligomers formed from epoxide, glyoxal and methylglyoxal[17]. Among these aqSOA components, oxalic acid, which is mainly formed from the glyoxal oxidation in cloud water, is the major aqSOA component[17]. The mechanism includes partitioning of glyoxal and methylglyoxal into water based on their effective Henry's law constants[21, 22].

The multiphase reaction scheme is used to predict aqSOA in aerosol water as in cloud water. Thus, aqSOA can be formed and lost in aged dust aerosols containing calcium nitrate in the base model. However, the solubility of glyoxal depends on the salt content in aerosol water. Salting-in effect on the effective Henry's law constant (2.7×10^{7} M atm^{-1})[23] was considered for nonreactive uptake of glyoxal on sulfate aerosol in the base model[16] but did not result in substantial aqSOA formation in supermicron aerosols (Supplementary Fig. 7A). Lin et al.[17] have already shown an increase in aqSOA by 8% only due to salting-in effects on the nonreactive uptake of glyoxal and methylglyoxal in aerosol water by an increase of 3 orders of magnitude for the effective Henry's law constants. As such, in the base simulations, aqSOA formation is represented in the model as a combination of the reactive uptake (on sulfate only) and bulk-phase (in all aerosol water) reactions[17]. The reactive uptake parameters for glyoxal (3.3×10^{-3}), for methylglyoxal (2.9×10^{-5}), and isoprene epoxide (2.9×10^{-3}) are used as

irreversible processes for aqueous sulfate aerosols only to form oligomer products. The reactive uptake coefficient for glyoxal is empirically derived for urban environments and is consistent with that for $(NH_4)_2SO_4$ seed aerosols in chamber experiments[24], which was 2 orders of magnitude higher than those determined on Gobi dust under dry dust surface[25]. The uptake coefficient of methylglyoxal was scaled to that of glyoxal by the ratio of their effective Henry's law constants[17].

Atmospheric SOA concentrations and oxygen-to-carbon (O/C) ratios predicated by several multiphase process schemes and reactive uptake coefficients in the base model have been evaluated against observations[17]. The use of reactive uptake method for sulfate aerosol (pathway (iv)) led to higher SOA than that predicted from nonreactive uptake. This worsened the model performance against observations when the model that does not include the reactive uptake reproduced the SOA concentration well but improved its performance when the model underestimated SOA[17]. In all cases, the base model with the reactive uptake resulted in higher O/C ratios than observations[26] because the O/C ratio for oligomers from glyoxal is assumed to be 1.5[17].

A resistor model was used to estimate the reactive uptake coefficients of glyoxal and methylglyoxal for sulfate/nitrate/ammonium aerosols and sea salt aerosols (mechanism (iv) above) following previous studies[27, 28]. Their results suggest that the uptake coefficients used in global models (i.e., 3.3×10^{-3} for glyoxal and 2.9×10^{-5} for methylglyoxal, respectively) are often too high than those calculated under realistic conditions. Indeed, the single reactive uptake coefficient for glyoxal, which was empirically derived for urban environments, led to an overestimation of SOA at two continental background stations (Supplementary Fig. 8). Therefore, in the improved model, we apply the resistor module in IMPCAT for an aqSOA formation via a reactive uptake of glyoxal and methylglyoxal on dust, combustion, and sea spray aerosols in addition to sulfate aerosols. This improved model calculates the reactive uptake coefficient, γ, of glyoxal, methylglyoxal, and isoprene epoxide to aqueous aerosols using the resistor model for laboratory-derived reactive uptake coefficients[27, 29]:

$$\frac{1}{\gamma} = \frac{1}{\alpha} + \frac{\omega}{4H^{*}RT\sqrt{k^{I}\,D_{aq}}}\left(\frac{1}{\coth q - 1/q}\right) \quad (1)$$

where α is the mass accommodation coefficient, ω denotes the gas phase thermal velocity of the organics, H^* is the effective Henry's law constant, R is the universal gas constant (0.082 l atm K^{-1} mol^{-1}), T is temperature (K), k^I is the first order loss rate in aqueous system, and D_{aq} is the aqueous-phase diffusion coefficient for the organics. R_p is the particle radius, and diffusion limitations in particle are also considered through the factor $q = R_p/l$, where l is the diffusion reactive length:

$$l = \left(\frac{D_{aq}}{k^{I}}\right)^{1/2} \quad (2)$$

In our calculations, $D_{aq} = 10^{-9}$ m^2 s^{-1} was used for small organics. $\alpha = 0.02$ was applied to estimate the mass accommodation coefficient for both glyoxal and methylglyoxal, while $\alpha = 0.1$ was used for isoprene epoxide. The irreversible aqueous-phase loss process for glyoxal and methylglyoxal is the reaction with OH radicals[30, 31], while that for isoprene epoxide includes acid-catalyzed ring opening reactions with nucleophiles (sulfate and nitrate ions) and acids (proton and bisulfate)[32]. The k^I values of glyoxal and methylglyoxal used in the IMPACT model are shown in Table S3 in Lin et al., (2014)[17], while those of isoprene epoxide are summarized in Table 1 in Marais et al. (2016)[33]. Improved model used the parameterized Henry's law constants for the salting-in effect of glyoxal[34] and salting-out effect of methylglyoxal[35]. Thus, the reactive uptake coefficients of glyoxal decrease with increasing liquid water concentrations (and thus relative humidity) due to salting in, whereas the opposite is true for methylglyoxal ("salts out")[28]. The salting effects on the Henry's constants were calculated for a mixed $(NH_4)_2SO_4$ and NH_4NO_3 aerosols and sea salt following Waxman et al[35].

$$\log\left(\frac{K_{H,\,w}}{K_{H,\,salt}}\right) = K_{s,(NH_4)_2SO_4}\, C_{(NH_4)_2SO_4} + K_{s,NH_4NO_3}\, C_{NH_4NO_3} \quad (3)$$

where $K_{H,\,salt}$ is the Henry's constant for the salt mixed system. $C_{(NH_4)_2SO_4}$ and $C_{NH_4NO_3}$ are the molality of $(NH_4)_2SO_4$ and NH_4NO_3, respectively. $K_{s,(NH_4)_2SO_4}$ and K_{s,NH_4NO_3} are the salting constants, respectively. The sum of sulfate and bisulfate was used to calculate $C_{(NH_4)_2SO_4}$.

$$\log\left(\frac{K_{H,\,w}}{K_{H,\,NaCl}}\right) = K_{s,NaCl}\, C_{NaCl} \quad (4)$$

where $K_{H,w}$ and $K_{H,NaCl}$ are the Henry's constants for pure water and salt-containing aerosols, respectively. C_{NaCl} is the NaCl molality concentration, and $K_{s,\,NaCl}$ is the salting constant.

Although it has been recommended to represent salting behavior based on the molality of sulfate, nitrate, and chloride[35], the Henry's constants for the conditions in the aged dust particles containing calcium nitrate and carbonate are not known. Indeed, the improved model underestimated SOA concentrations during high-concentration events, likely due to the salting behavior under ambient aerosol conditions, which they typically contain a mixture of salts (Supplementary Fig. 8). Currently, there is a lack of laboratory data to constrain such salting behavior. For this reason, we evaluated the effects of salting effects for the reactive uptake on aqSOA mass concentrations. We examined the sensitivity of reactive uptake coefficients for glyoxal (and methylglyoxal) to the salting-in (salting-out) effects. Sensitivity simulation to the improved model used the measured Henry's law constants of glyoxal (2.7×10^7 M atm^{-1})[23], methylglyoxal (3.7×10^3 M atm^{-1})[36] and isoprene epoxide (3×10^7 M atm^{-1})[37] (Fig. 3A and Supplementary Fig. 8C and 8D). In the sensitivity simulations, the contribution of aqSOA formed on supermicron aerosols to total SOA in Crete (45±4%) is higher than observations

(see Supplementary Fig. 9). This suggests that the activity of salts modifies the effective Henry's law coefficients and thus influences aqSOA formation. To test this further statistically, we have applied a linear least squares regression to the observed WSOC fractions in supermicron aerosols with model results for base, sensitivity, and improved simulations (Supplementary Fig. 8). The results show significant improvement in coefficient of determination (R2) from 0.01 to 0.78 and 0.84 for base, sensitivity, and improved simulations, respectively. The regression slope from the improved simulations is the closest to the unity. The overall globally and annually averaged SOA concentrations are similar between the base model (35 μg m^{-3}) and the optimized model (36 μg m^{-3}), partly because the lower concentrations in submicron particles are compensated by higher concentration in supermicron particles to some extent.

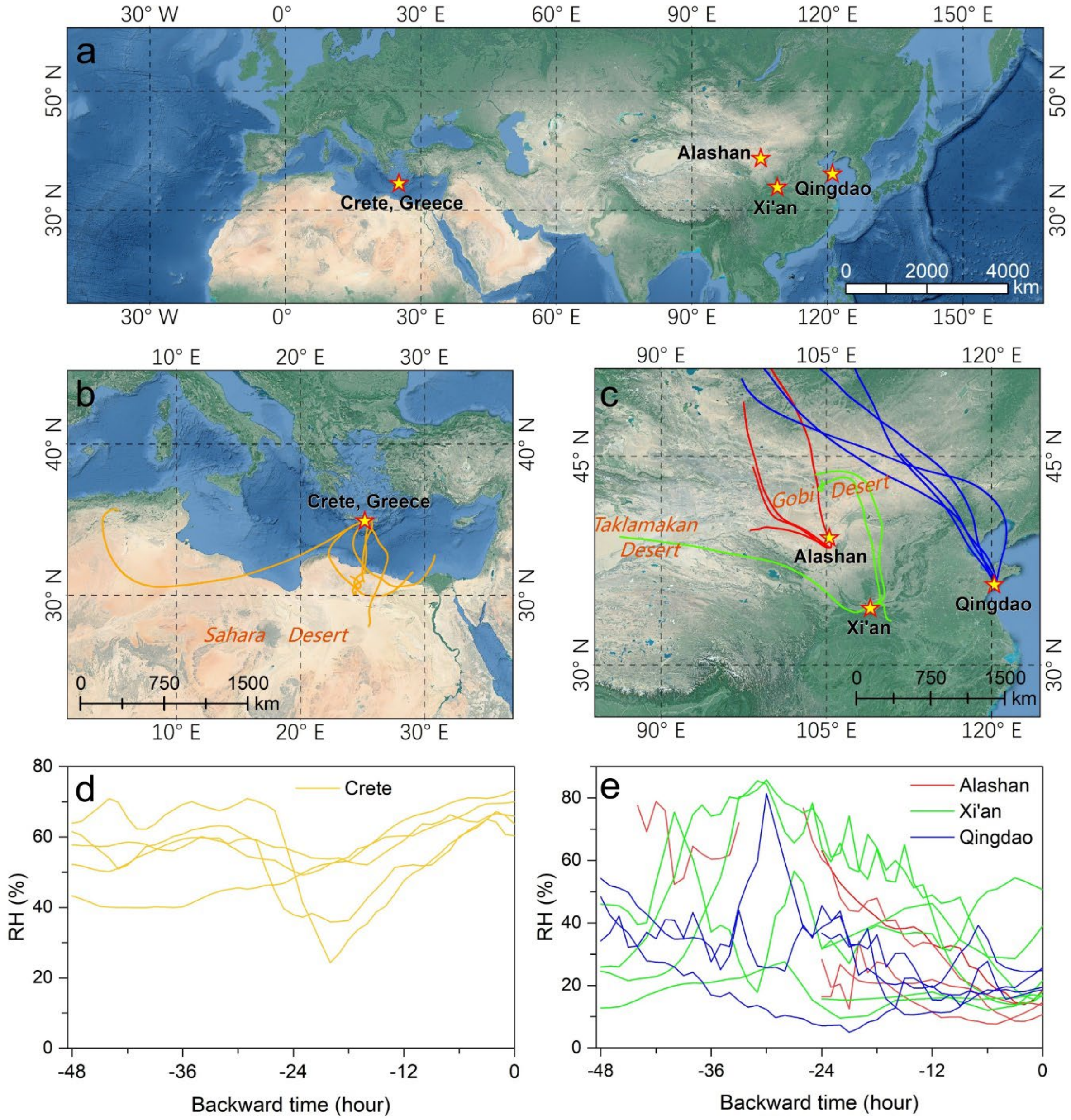

Figure S1 The locations of the aerosol sampling sites in this study. A, Crete (a downwind site of Sahara dust source region), Alashan (near Tengger Desert), Xi'an city (downwind city of Asia dust source region, and Qingdao city (a coastal city of East Asia). **B-C**, 48 hours backward trajectories of air masses during the dust events at all sampling sites. **D-E**, Relative humidity (RH) in the air masses along the trajectories during all studied dust events. The RHs were 20-70% during the African dust storms and 10-80% during Asian dust storms.

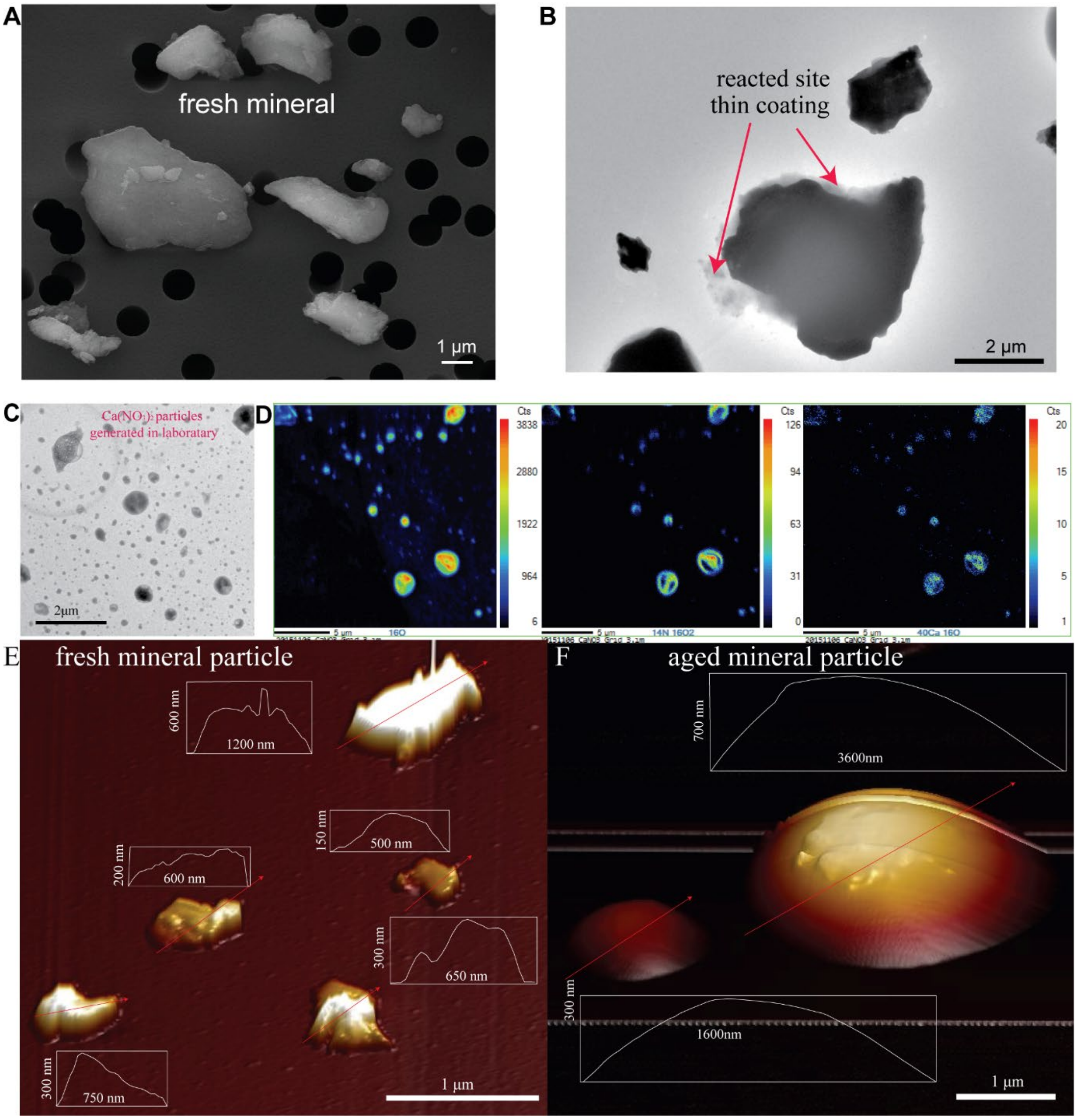

Figure S2 Morphology of fresh and aged dust particles collected at Alashan and NanoSIMS ion-intensity maps of $Ca(NO_3)_2$ particles generated in laboratory on TEM grids. A) fresh mineral dust particles collected during dust periods. These particles have irregular shapes with no identified coating; **B)** partly-reacted dust particles collected after dust days (red arrow indicates reacted sites).; **C)** laboratory generated $Ca(NO_3)_2$ particles on the substrate; **D)** NanoSIMS ion map of O^-, NO_2^-, and CaO^- ions in a laboratory-generated pure $Ca(NO_3)_2$ particle; **E**) Tomography of fresh mineral dust particles from AFM; **F**) Tomography of aged mineral dust particles.

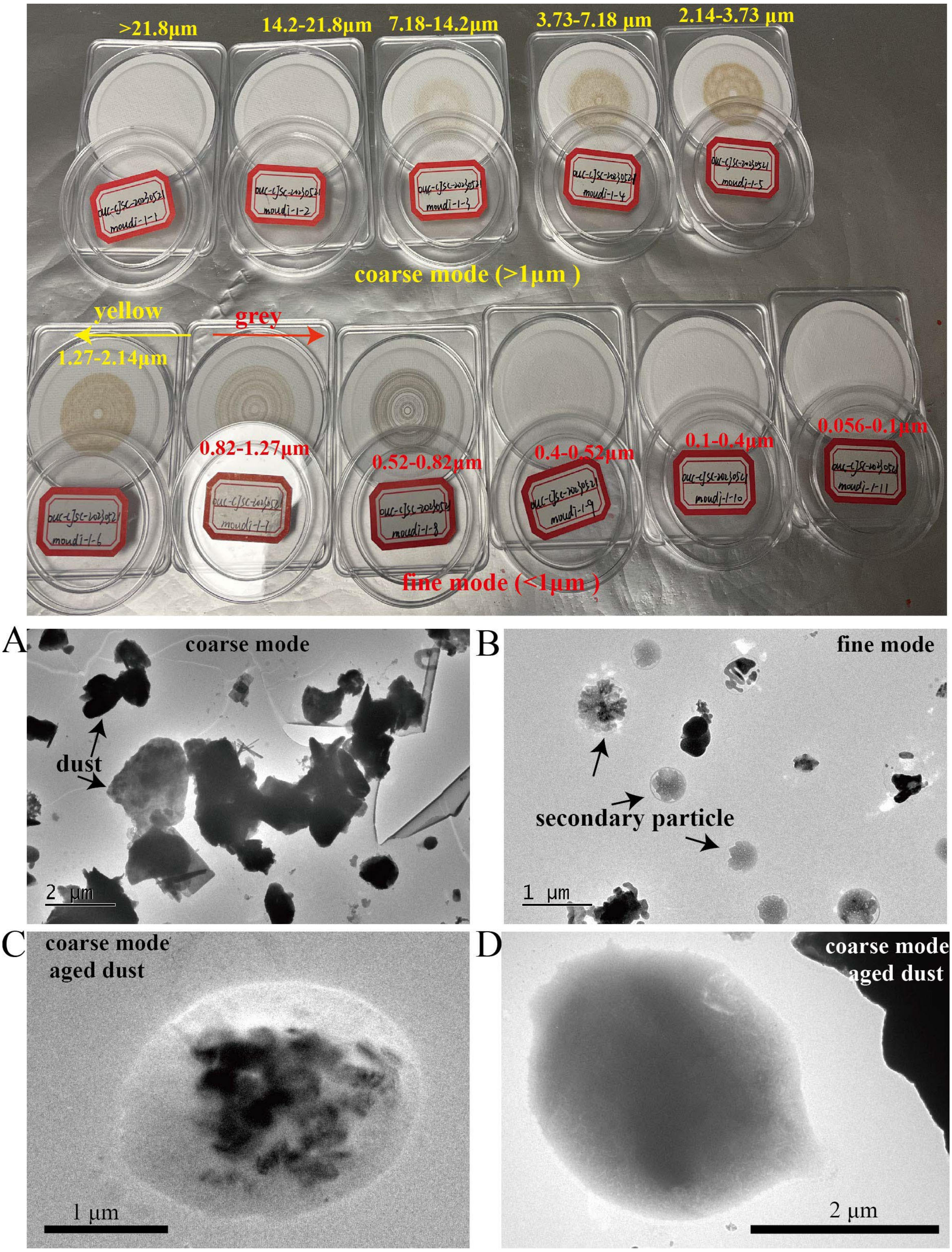

Figure S3 The classification of submicron and supermicron particles in size-resolved particles. The filters in supermicron particles display yellow color but the filters in submicron sizes show grey color. The low magnification TEM images show that supermicron particles mostly contain dust particles (A) and submicron particles mostly contain secondary particles (B). Also, we detected some aged dust particles in supermicron size (C, D).

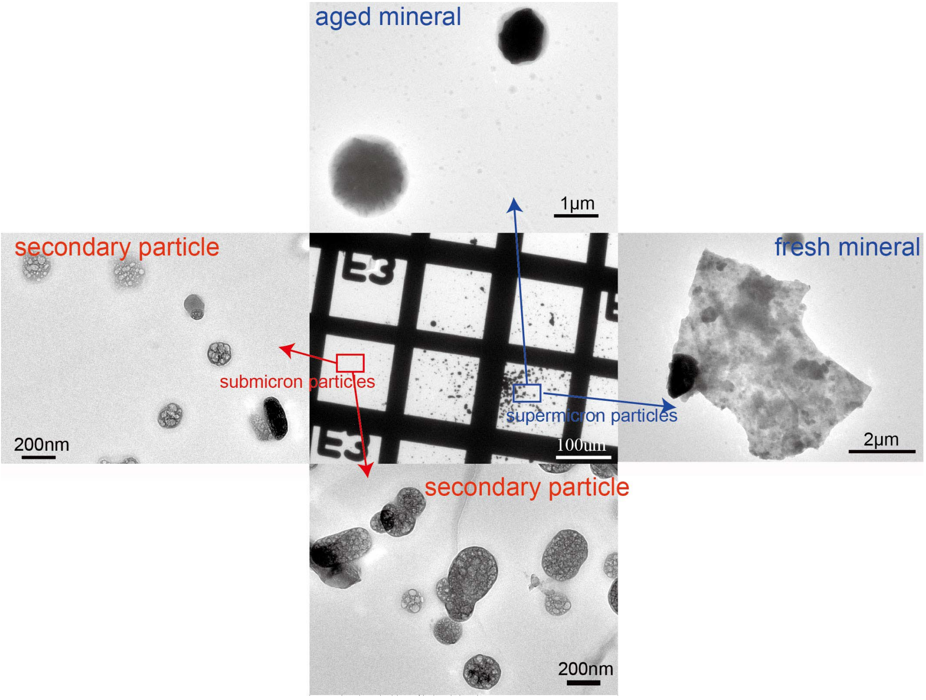

Figure S4 Morphology of submicron and supermicron particles collected at Alashan. Submicron particles are dominated by secondary particles and supermicron particles are dominated by fresh and aged dust particles.

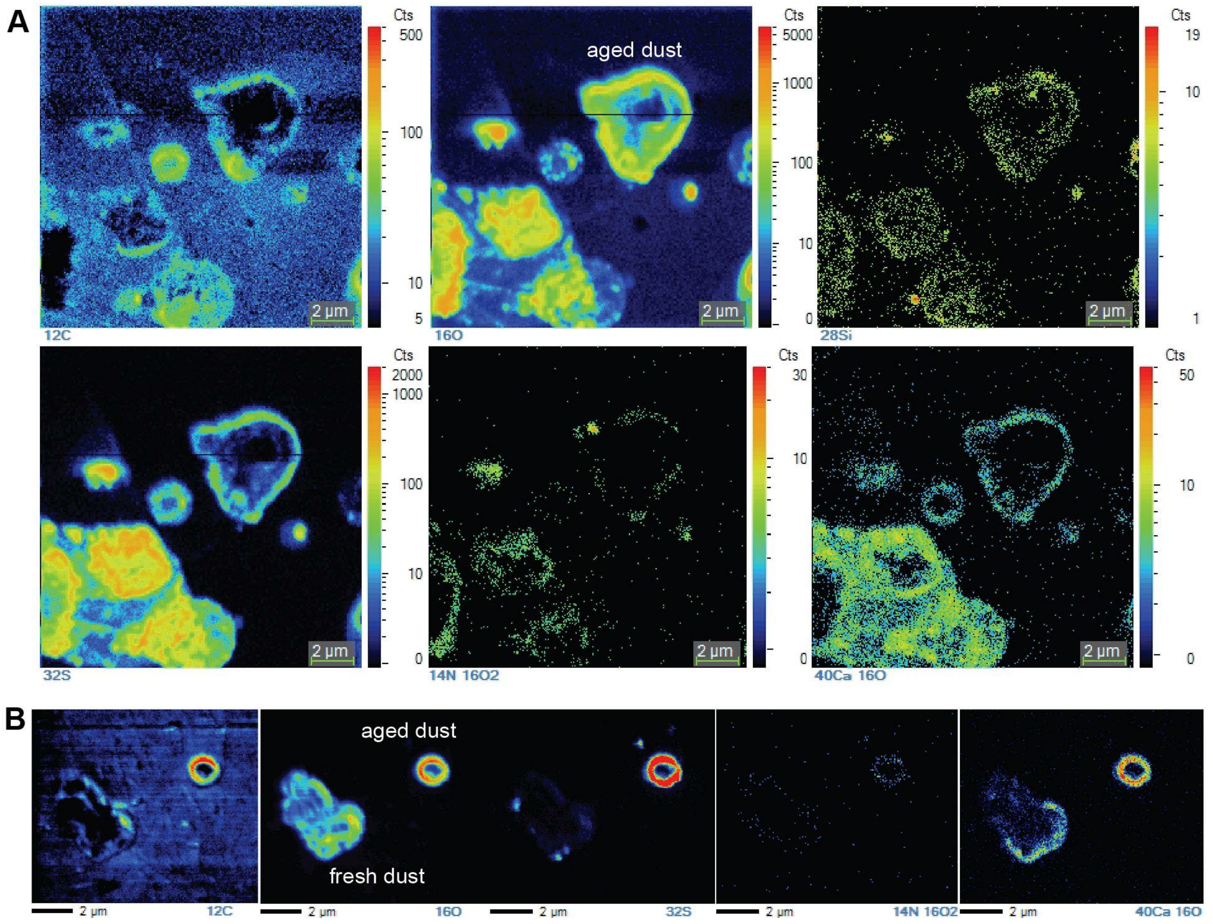

Figure S5 NanoSIMS ion-intensity maps of aged mineral dust particles collected at Alashan. **A)** $^{12}C^-$, $^{12}O^-$, $^{28}Si^-$, $^{32}S^-$, $^{14}N^{16}O_2^-$, and $^{40}Ca^{16}O^-$ ion mapping of an aged dust particle. **B)** NanoSIMS maps of $^{12}C^-$, $^{12}O^-$, $^{32}S^-$, $^{14}N^{16}O_2^-$, and $^{40}Ca^{16}O^-$ ions in fresh and aged dust particles. $^{2}C^-$, $^{12}O^-$, $^{14}N^{16}O_2^-$, and $^{40}Ca^{16}O^-$ ion mapping occur in the Ca-rich coatings.

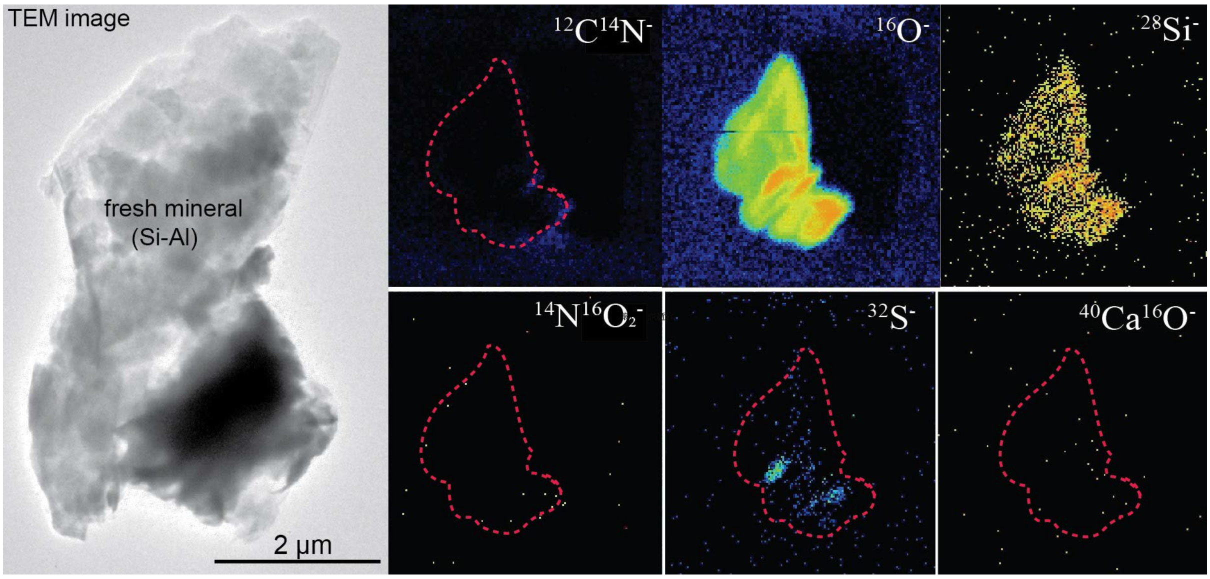

Figure S6 TEM image and NanoSIMS ion-intensity maps of a **fresh dust particle collected during Asian dust event in Alashan.** NanoSIMS shows $^{12}C^{14}N^-$, $^{12}O^-$, $^{28}Si^-$, $^{14}N^{16}O_2^-$, $^{32}S^-$, and $^{40}Ca^{16}O^-$ ion mapping of a fresh dust particle. CN^- signal was not detectable in the particle.

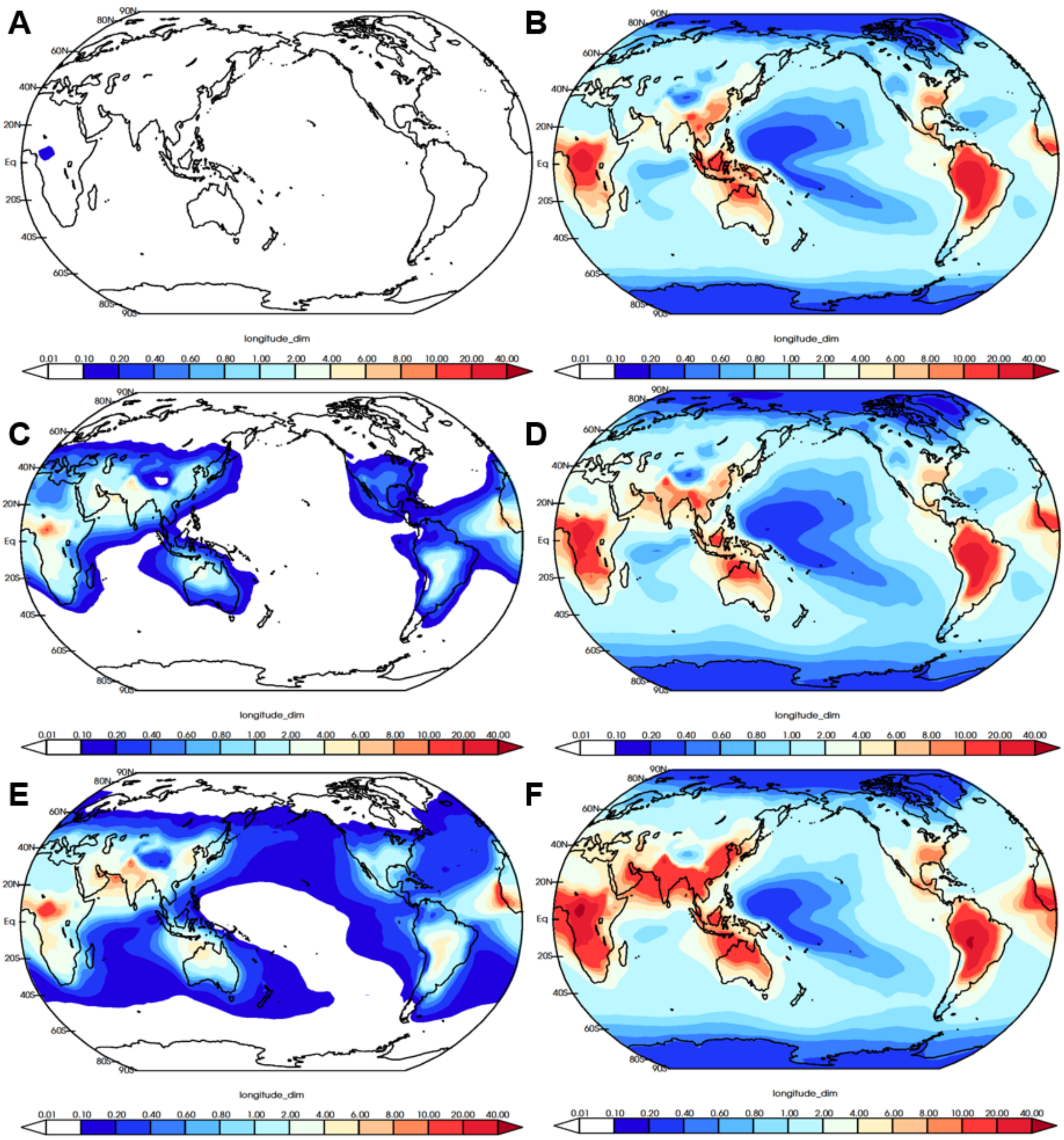

Figure S7 Global aqSOA in supermicron dust and SOA in all aerosols (μg m^{-3}) near the ground in the base model (A, B), improved model (C, D) and sensitivity case (E, F), respectively, for the year of 2017 simulated by the IMPACT model. In the base model, aqSOA formed in supermicron dust **(A, B)** is negligible. In the improved model **(C, D)**, aqSOA concentrations on supermicron dust are high in the downstream region of dust outflow. In the sensitivity simulation to the improved model, aqSOA concentrations on supermicron dust are higher than those in the improved model **(E, F)**.

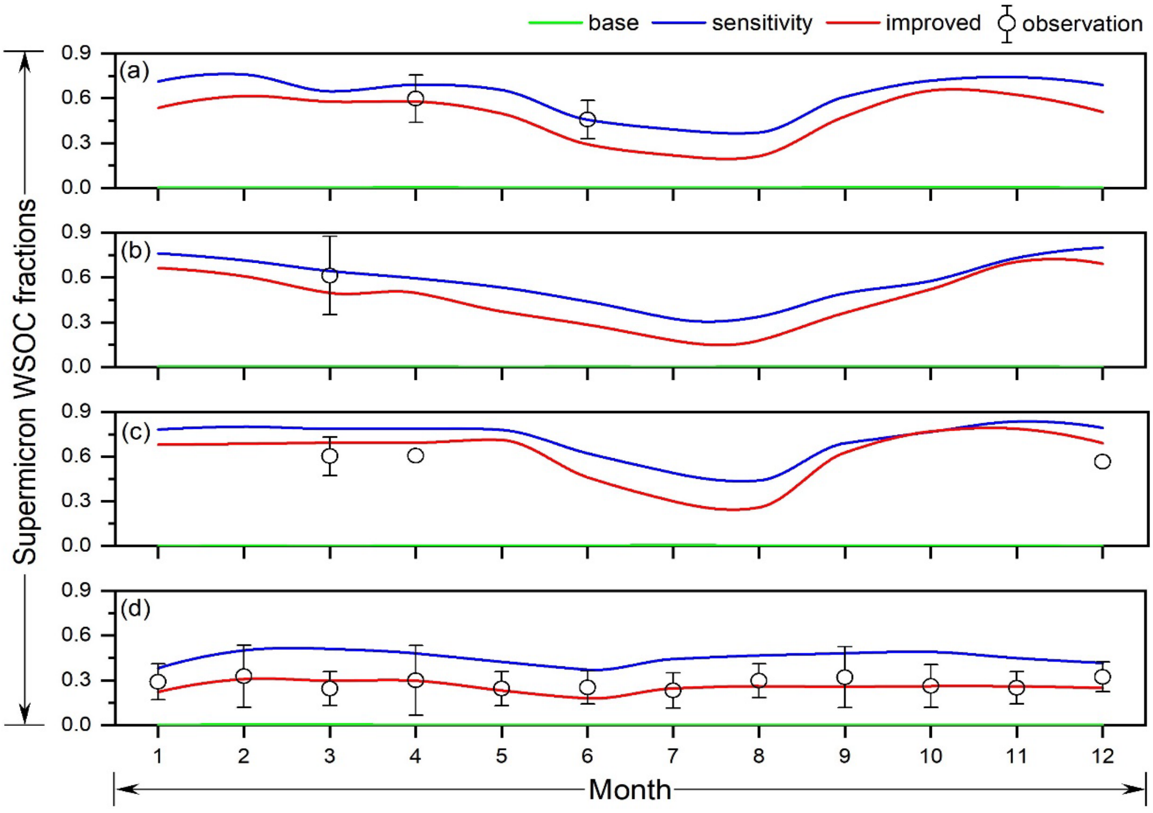

Figure S8 Comparison of modeled versus observed WSOC fractions in supermicron aerosols at Alashan (A), Xi'an (B), Qingdao (C) and Crete (D), respectively. The green, blue, and red color line represent the results from the base, sensitivity, and improve model simulations. The monthly averages and standard deviations were calculated from the daily output in 2017.

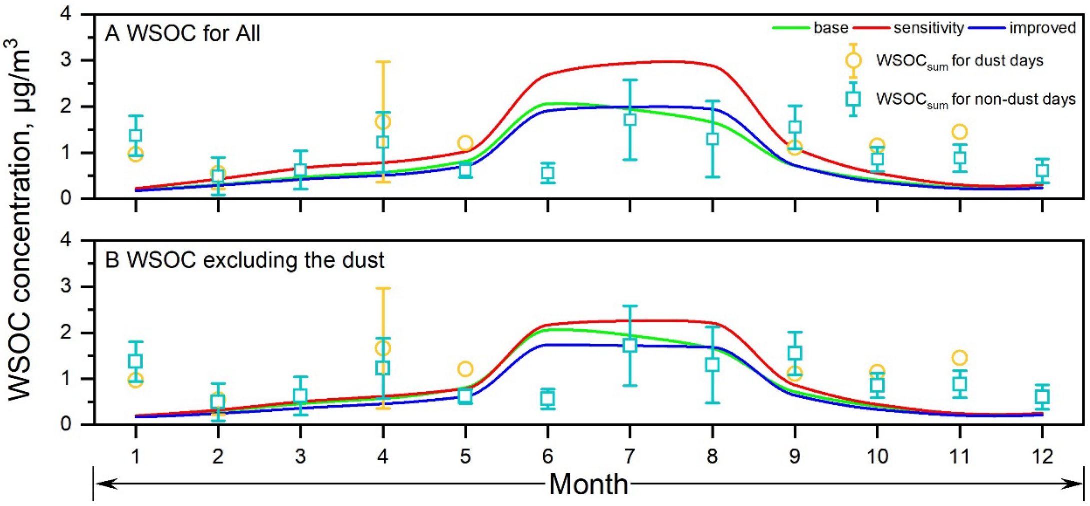

Figure S9 Comparison of the $WSOC_{sum}$ during the dust and non-dust days at Crete. (A) all the WSOC, (B) WSOC excluding the dust. The color lines in (A) and (B) represent the simulated WSOC for the base (green), sensitivity (red), and improved (blue) models, respectively.

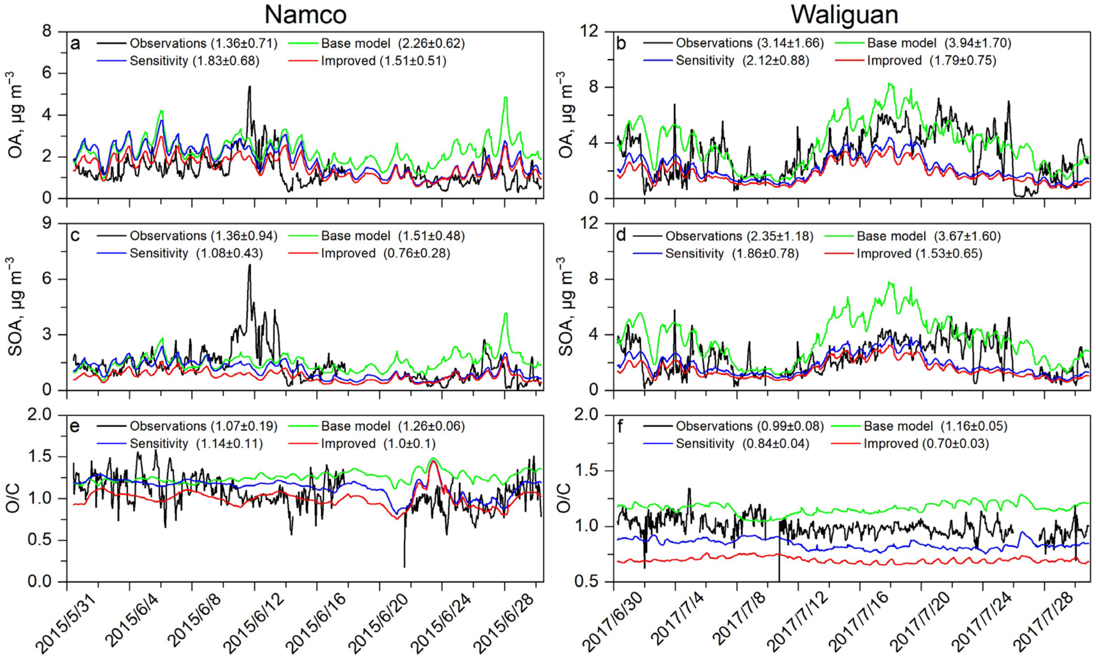

Figure S10 Time series of the concentrations of submicron organic aerosol (A, B), SOA (C, D), and O/C (E, F) at NamCo and Waliguan sites, from 31 May to 30 June 2015 and from 1 July to 31 July 2017: measured, base (green), improved (red) and sensitivity (blue) simulations. The observed data were from the Xu et al. (37).

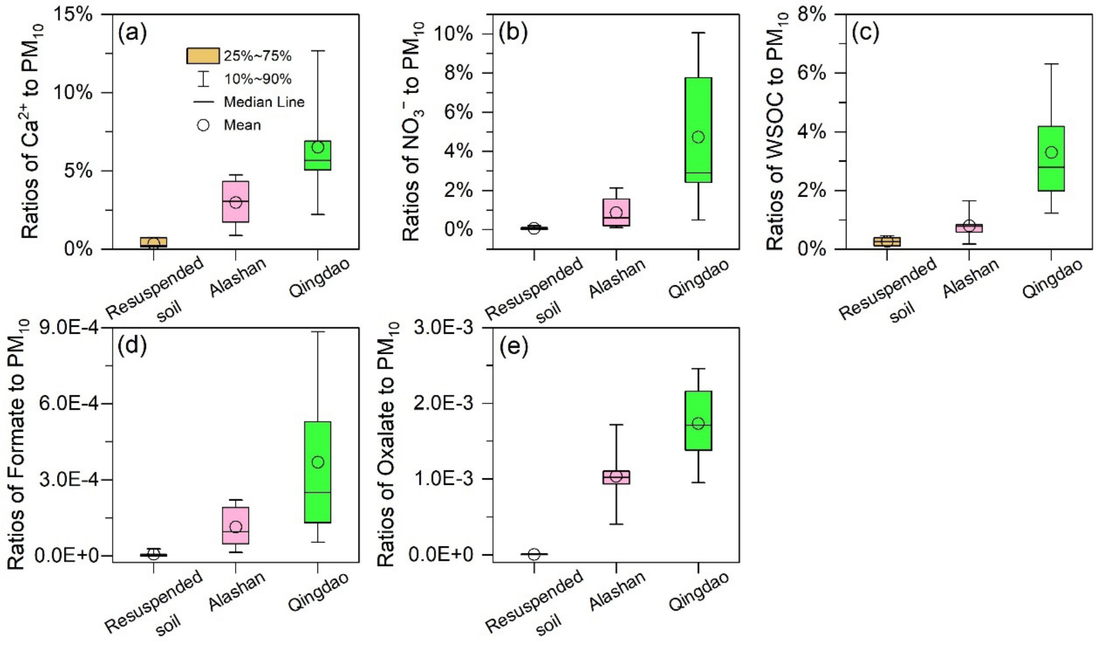

Figure S11 Percentage contribution of Ca^{2+} (A), NO_3^- (B), WSOC (C), Formate (D), Oxalate (E) to PM_{10} mass from resuspended soils collected in Tengger Desert, and ambient PM_{10} from Alashan, and Qingdao during the dust periods. The Alashan site locates about 200 km downwind of the central Tengger desert, and Qingdao locates about 1200 km downwind of Tengger desert. The dust samples in Alashan were collected in April, 2021 and those in Qingdao were collected during the same dust events in March-April, 2023 (see methods).

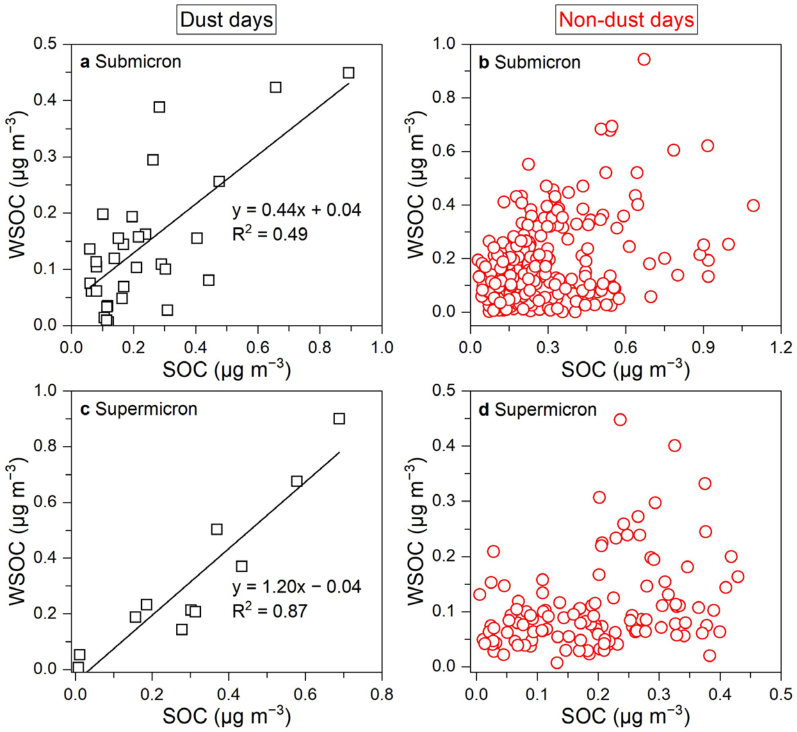

Figure S12 Correlations between secondary organic carbon (SOC) and water-soluble organic carbon at submicron and supermicron particles on dusty days (a and c) and non-dusty days (b and d), respectively, in Crete, Greece. Here, SOC (SOC = OC−$(OC/EC)_{pri}$ * EC) was referring to the method provided by Lim and Turpin (2002) and assumed the lowest OC/EC ratio as OC/EC_{pri}.

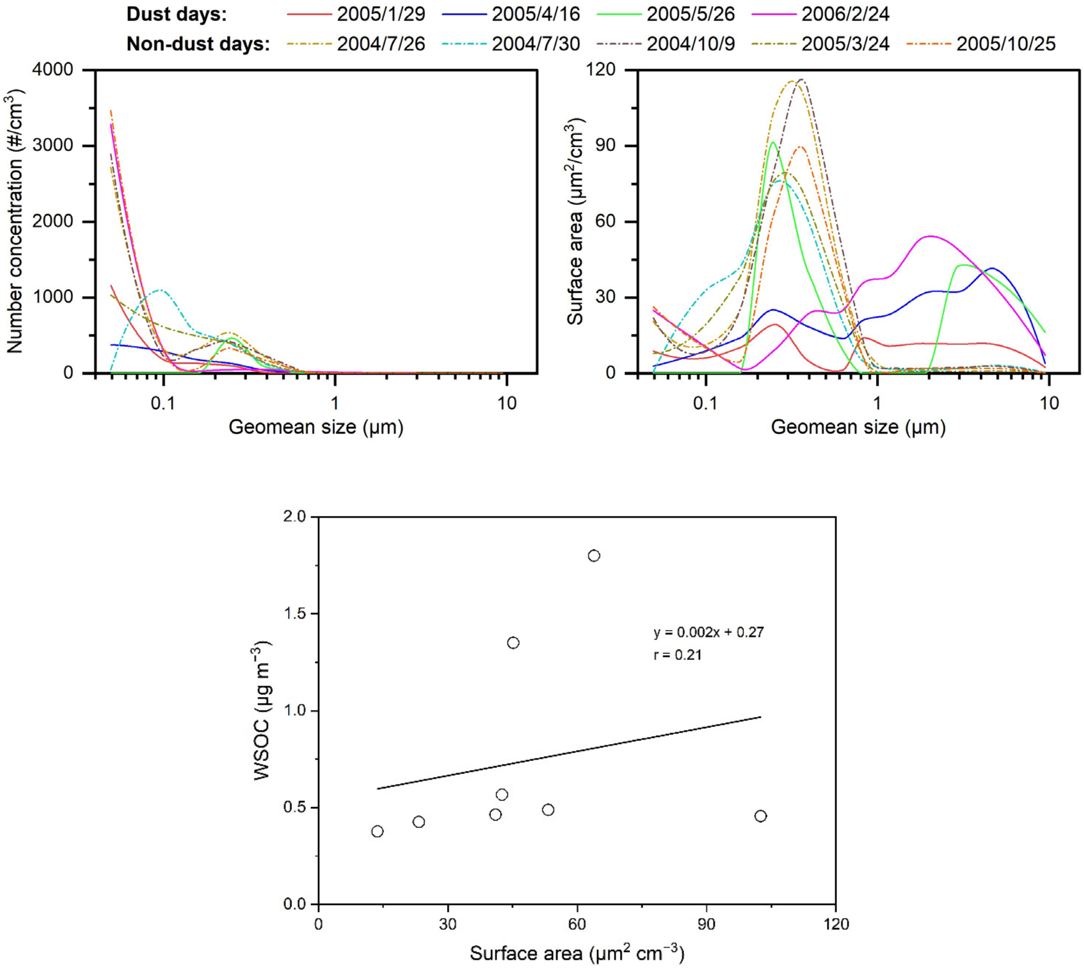

Figure S13 Size distribution of (A) particle number and (B) surface area, and C) the correlations between WSOC and surface area in supermicron particles ($D_p > 1$ μm) during dust at Crete site where is downwind of the African dust storm. The particle size from 40 nm-10 μm was calculated by particle mass. The surface areas in the supermicron particles during dust days are much larger than those during non-dust days.

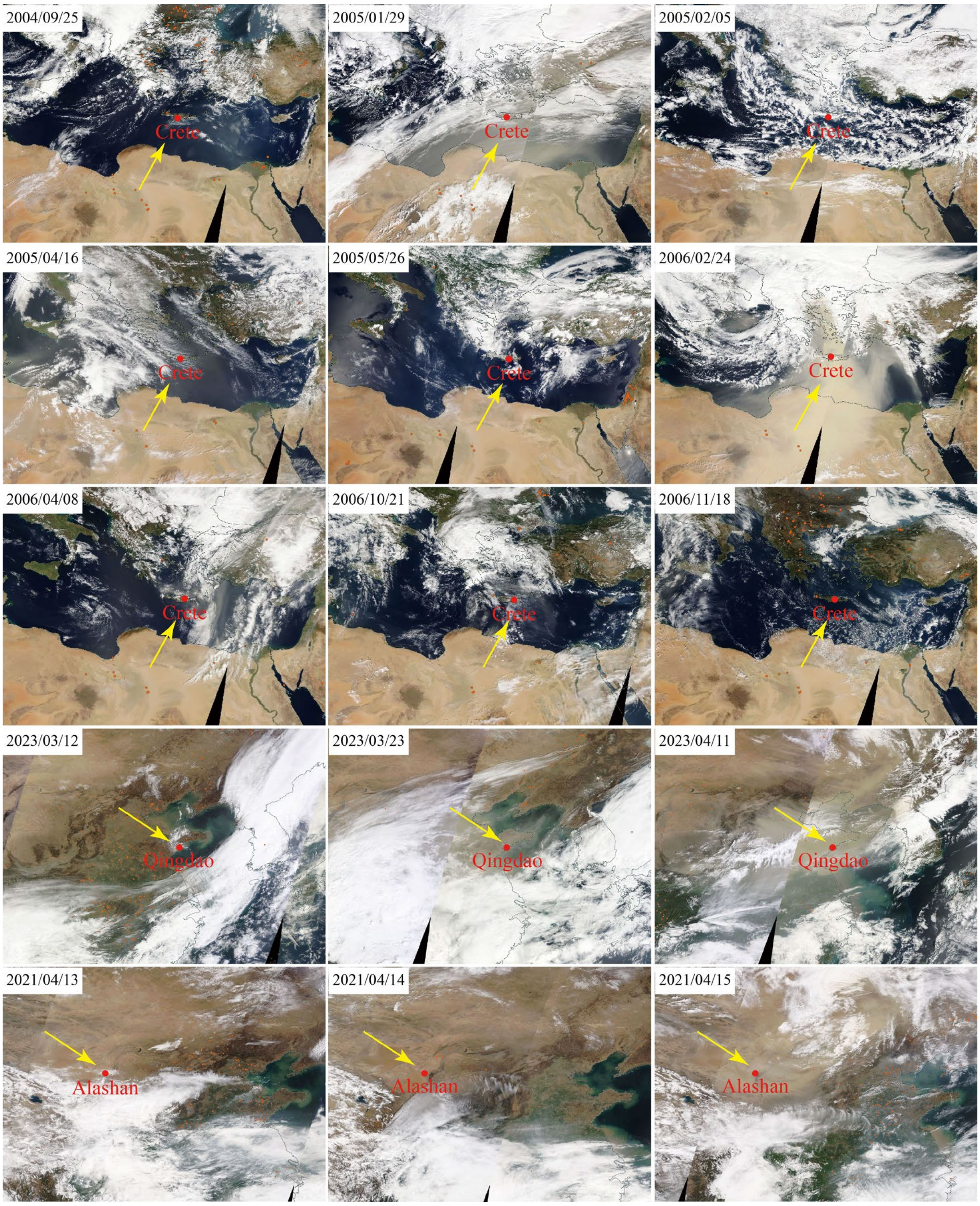

Figure S14 Satellite images in all the sampling dust day. There were no evidence of dust mixing with clouds before the dust storm arrived at the sampling site. Yellow arrows represent dust transport direction. The red spots represent the biomass burning.

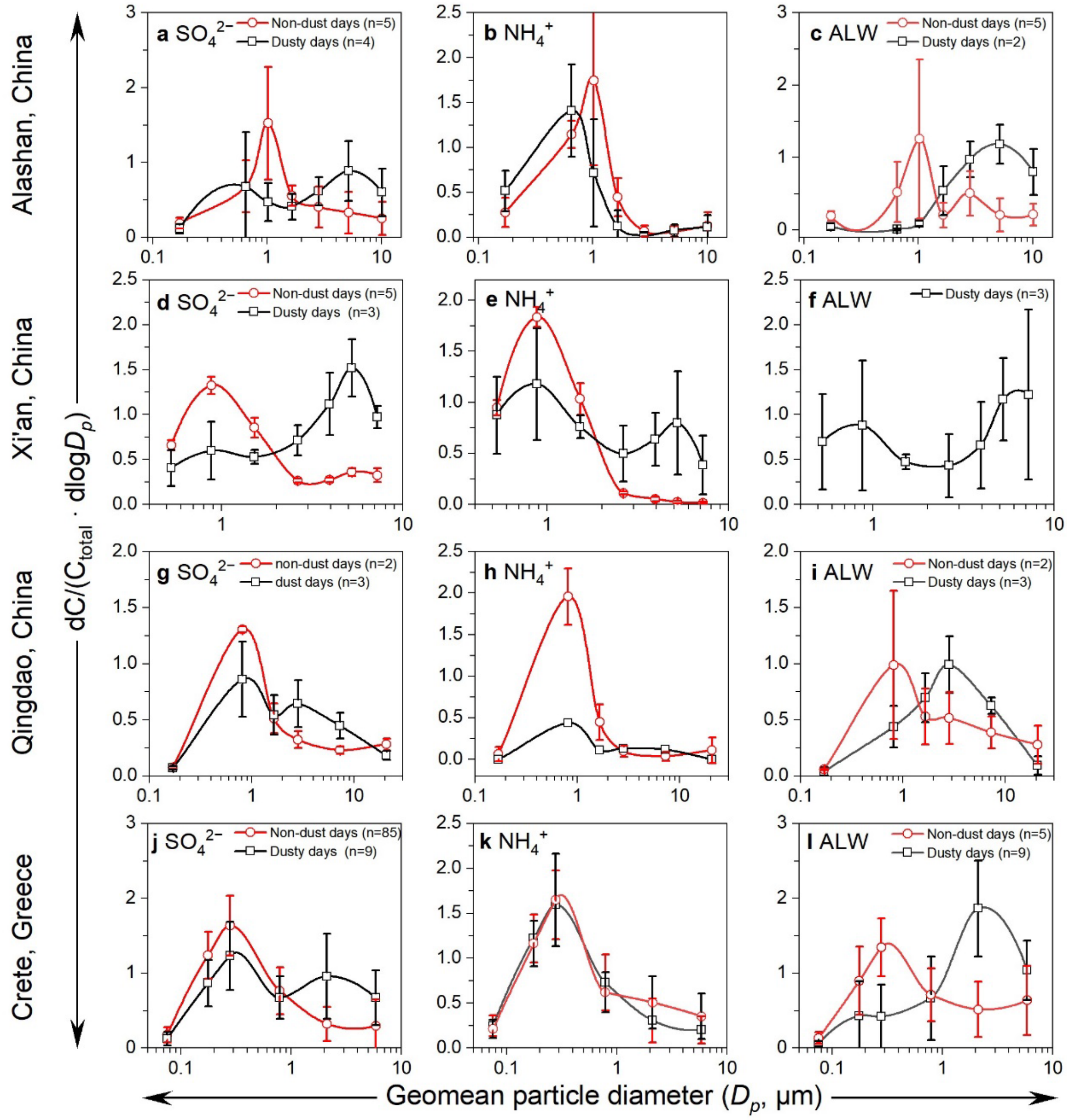

Figure S15 Size distribution of SO_4^{2-}, NH_4^+, and aerosol liquid water (ALW) at Alashan (A, B, and C), Xi'an (D, E, and F), Qingdao (G, H, and I), and Crete (J, K, and L). ALW was calculated by ISORROPIA model. The above data are normalized based on the $dC_i/dC_{i\text{-all}}$ (see Methods)..

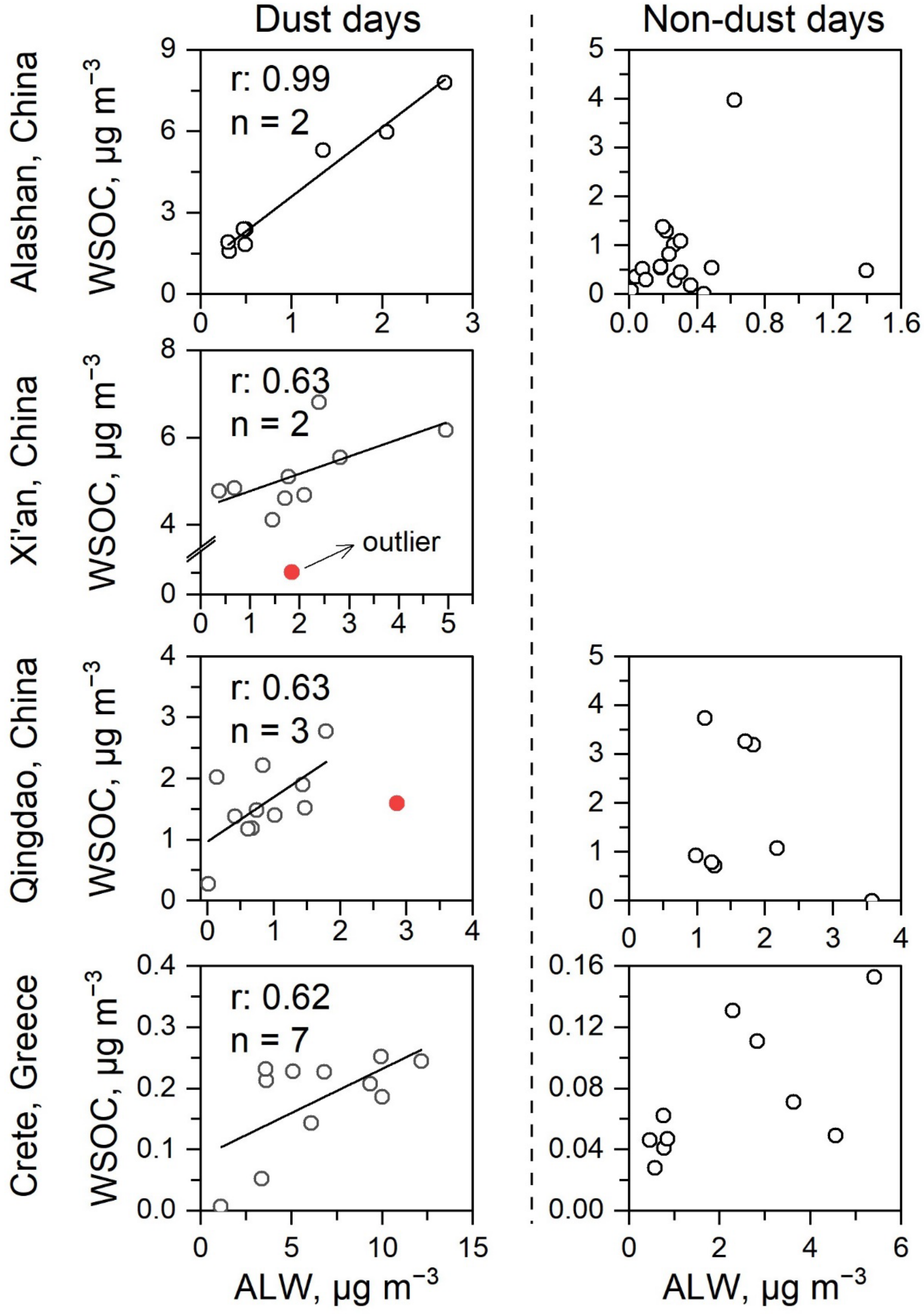

Figure S16 Relationships between ALW and WSOC in the supermicron particles during dust and non-dust days in Alashan, Xi'an, Qingdao and Crete, respectively.

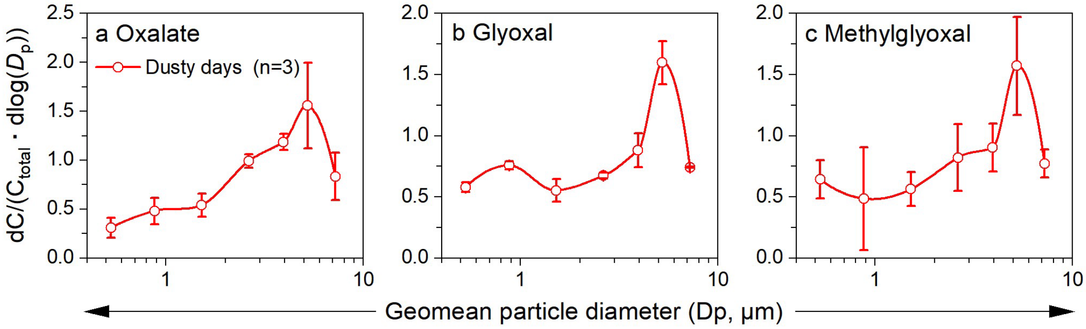

Figure S17 Size distribution of oxalate, Glyoxal, and Methylglyoxal (A, B, C) during Asian dust storm at Xi'an. The above data are normalized based on the $dC_i/dC_{i\text{-all}}$ (see Methods).

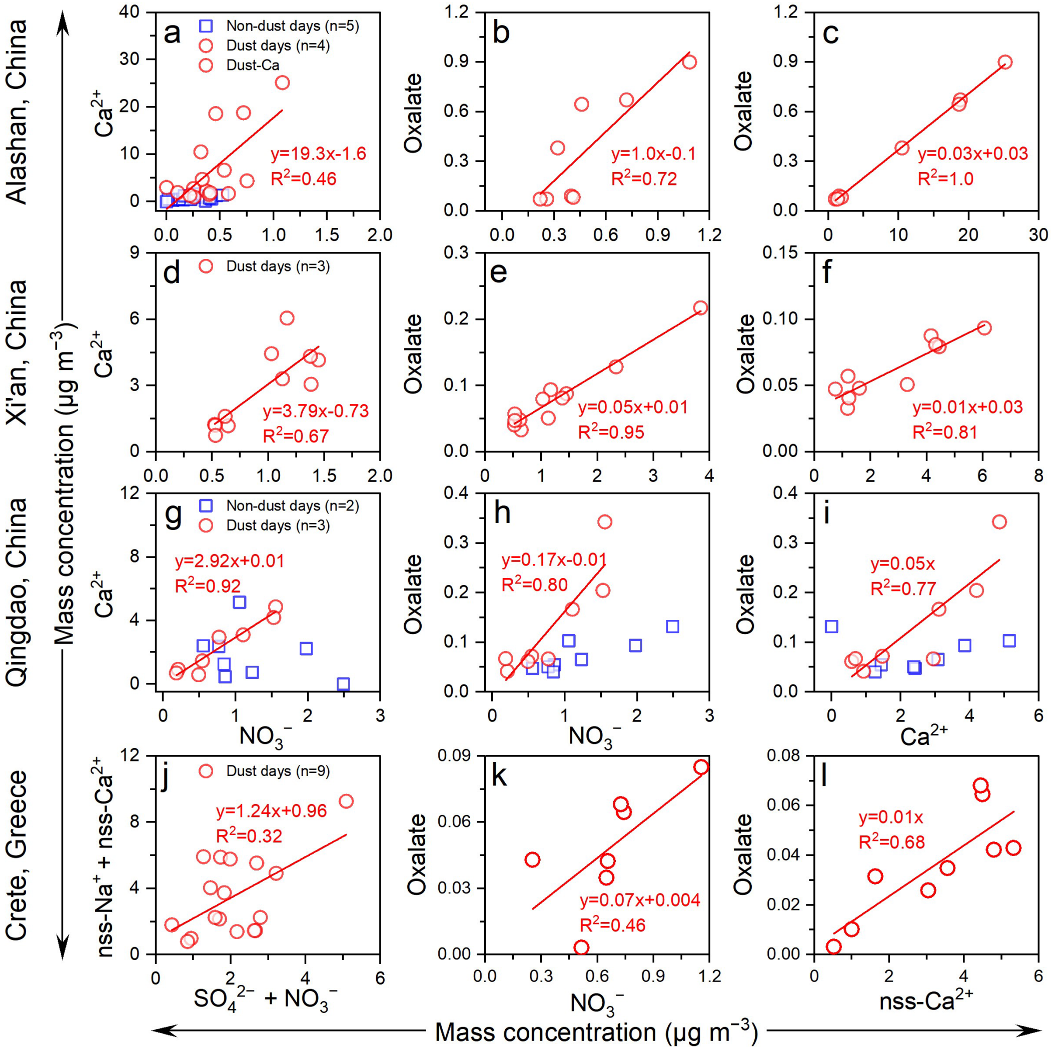

Figure S18 Linear fit regression for NO_3^- vs. Ca^{2+}, NO_3^- vs. oxalate, Ca^{2+} vs. oxalate, Ca^{2+} vs. WSOC, Ca^{2+} vs. ALW at Alashan, Xian, Qingdao and Crete, respectively. Most of these species have good correlation. Note that the two data points in Fig. 6k correspond to relatively weak dust (Ca^{2+} less than 2 μg m^{-3}) in the Crete sample and are not included here.

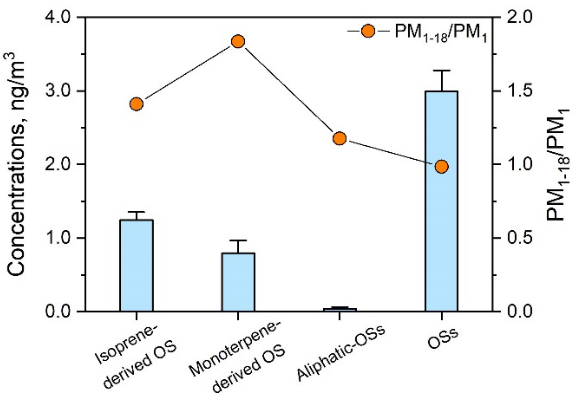

Figure 19 Average concentrations (in ng m^{-3}) of isoprene-derived, monoterpene-derived, aliphatic, and total OSs in two size-resolved dust samples collected in Qingdao and their concentration ratios in supermicron vs submicron particles quantified by UPLC-ESI-QToFMS.

Table S1 DRH at 25 °C for the selected Water-Soluble Organic Acids and Ca-containing compounds (cited from[38, 39])

Species	DRH(%)
oxalic acid	97.3
succinic acid	98.8
glutaric acid	88.0−88.5
$Ca(NO_3)_2$	8%
Calcium oxalate	>90%
Calcium sulfate	>95%
Sodium oxalate	>90%

Table S2 Information of three types of mineral dust particles at three sampling sites

sampling dust stage	mineral particles		number	percentage	Size (□ m) mean ±stdev	Sphericity mean ±stdev
After dust storm and non-dust storm (Alashan)	fresh		446	65%	1.84.±1.37	0.49±0.20
	Aged	core-shell	164	24%	1.92±1.07	0.75±0.22
		no core	60	9%	1.68±0.84	0.88±0.19
	$CaSO_4$-containing		18	2%	1.30±1.31	0.28±0.23
During dust storm (Alashan)	fresh		259	100%	0.653±0.51	0.42±0.21
After dust storm (Qingdao)	fresh		57	49%	3.29±1.55	0.47±0.20
	Aged	core-shell	27	23%	2.72±1.15	
		no core	5	4%	1.56±0.59	
	$CaSO_4$-containing		27	23%	2.01±0.98	0.35±0.20

Table S3 Mass ratios of ALW_Ca$(NO_3)_2$/ALW_{total} simulated by ISORROPIA II thermodynamic model in Alashan, China

Type	Date	Diameter (μm)	ALW_{total} (μg m^{-3})	ALW_Ca$(NO_3)_2$ (μg m^{-3})	ALW_Ca$(NO_3)_2$ /ALW_{total}	WSOC (μg m^{-3})
Dust days	2021/4/12~4/14	1.27-2.14	0.312	0.117	0.376	1.584
		2.14-3.73	0.487	0.462	0.949	1.832
		3.73-7.18	0.497	0.449	0.903	2.409
		7.18-14.2	0.300	0.244	0.812	1.910
	2021/4/14~4/15	1.27-2.14	0.481	0.472	0.980	2.406
		2.14-3.73	1.342	1.351	1.000	5.310
		3.73-7.18	2.717	2.689	0.990	7.787
		7.18-14.2	2.111	2.047	0.970	5.979
Non-dust days	2019/6/24~6/25	1.27-2.14	-	-	-	1.713
		2.14-3.73	0.487	0.478	0.981	0.546
		3.73-7.18	0.263	0.234	0.890	1.008
		7.18-14.2	0.303	0.162	0.535	1.083
	2019/6/28~6/29	1.27-2.14	0.186	0.174	0.933	0.543
		2.14-3.73	0.364	0.350	0.960	0.184
		3.73-7.18	0.271	0.253	0.931	0.291
		7.18-14.2	0.186	0.152	0.817	0.569
	2019/6/29~6/30	1.27-2.14	0.040	0.000	0.000	0.360
		2.14-3.73	0.080	0.072	0.892	0.520
		3.73-7.18	0.013	0.000	0.000	0.081
		7.18-14.2	0.101	0.054	0.533	0.302
	2021/4/5~4/6	1.27-2.14	0.220	0.000	0.000	1.297
		2.14-3.73	0.619	0.148	0.240	3.972
		3.73-7.18	0.304	0.181	0.596	0.447
		7.18-14.2	0.199	0.147	0.741	1.379
	2021/4/6~4/8	1.27-2.14	1.394	2.547	1.000	0.487
		2.14-3.73	-	-	-	0.146
		3.73-7.18	0.441	0.367	0.830	0.009
		7.18-14.2	0.236	0.212	0.896	0.817

Table S4 Mass ratios of ALW_$Ca(NO_3)_2$/ALW_{total} simulated by ISORROPIA II thermodynamic model in Xi'an, China

Type	Date	Diameter (μm)	ALW_{total} (μg m^{-3})	ALW_$Ca(NO_3)_2$ (μg m^{-3})	ALW_$Ca(NO_3)_2$ /ALW_{total}	WSOC (μg m^{-3})
Dust days	2009/3/24	5.8-9.0	4.951	4.910	0.992	6.166
		4.7-5.8	1.445	1.531	1.000	4.116
		3.3-4.7	0.683	0.878	1.000	4.847
		2.1-3.3	0.369	0.062	0.168	4.775
		1.1-2.1	1.845	1.831	0.993	0.520
	2011/3/29	5.8-9.0	2.814	2.701	0.960	5.549
		4.7-5.8	1.775	1.675	0.944	5.101
		3.3-4.7	2.392	2.255	0.942	6.807
		2.1-3.3	2.088	1.931	0.925	4.693
		1.1-2.1	1.701	1.445	0.849	4.610
	2011/3/30	5.8-9.0	0.224	-	-	
		4.7-5.8	0.224	-	-	
		3.3-4.7	0.224	-	-	
		2.1-3.3	0.224	-	-	
		1.1-2.1	0.415	-	-	

Table S5 Mass ratios of ALW_$Ca(NO_3)_2$/ALW_{total} simulated by ISORROPIA II thermodynamic model in Qingdao, China

Type	Date	Diameter (μm)	ALW_{total} (μg m^{-3})	ALW_$Ca(NO_3)_2$ (μg m^{-3})	ALW_$Ca(NO_3)_2$/ ALW_{total}	WSOC (μg m^{-3})
Dust days	2023/3/11~ 3/12	14.2-30	0.412	0.412	1.000	1.378
		3.73-14.2	2.855	2.855	1.000	1.599
		2.14-3.73	1.471	1.471	1.000	1.524
		1.27-2.14	0.741	0.741	1.000	1.485
	2023/3/23~ 3/24	14.2-30	0.140	0.140	1.000	2.024
		3.73-14.2	1.785	1.490	0.834	2.773
		2.14-3.73	1.435	1.094	0.762	1.904
		1.27-2.14	0.837	0.546	0.652	2.215
	2023/4/11~ 4/11	14.2-30	0.010	0.010	1.000	0.277
		3.73-14.2	1.014	1.014	1.000	1.403
		2.14-3.73	0.669	0.669	1.000	1.186
		1.27-2.14	0.616	0.616	1.000	1.174
Non-dust days	2022/12/9	14.2-30	1.115	0.140	0.126	3.735
		3.73-14.2	3.571	0.000	0.000	0.000
		2.14-3.73	1.831	1.496	0.817	3.186
		1.27-2.14	1.710	0.000	0.000	3.260
	2023/3/25~ 3/26	14.2-30	0.985	0.493	0.500	0.926
		3.73-14.2	2.173	1.709	0.787	1.072
		2.14-3.73	1.256	0.886	0.706	0.714
		1.27-2.14	1.221	0.501	0.411	0.791

Table S6 Mass ratios of ALW_Ca(NO_3)$_2$/ALW_{total} simulated by ISORROPIA II thermodynamic model in Crete, Greece

Type	Date	Diameter (μm)	ALW_{total} (μg m^{-3})	ALW_Ca(NO_3)$_2$ (μg m^{-3})	ALW_Ca(NO_3)$_2$ /ALW_{total}	WSOC (μg m^{-3})
Dust days	2004/9/25	1.66-2.68	3.437	3.041	0.885	0.370
		4.08-8.39	4.665	4.486	0.962	0.503
	2005/1/29	1.66-2.68	3.609	3.460	0.959	0.213
		4.08-8.39	-	-	-	0.188
	2005/2/5	1.66-2.68	3.348	3.280	0.980	0.052
		4.08-8.39	1.117	0.874	0.782	0.007
	2005/4/16	1.66-2.68	2.471	2.012	0.814	0.900
		4.08-8.39	3.156	2.634	0.835	0.675
	2005/5/26	1.66-2.68	68.255	66.180	0.970	0.283
		4.08-8.39	133.133	129.744	0.975	0.244
	2006/2/24	1.66-2.68	5.071	4.854	0.957	0.228
		4.08-8.39	3.581	3.583	1.000	0.232
	2006/4/8	1.66-2.68	9.990	9.389	0.940	0.186
		4.08-8.39	6.810	6.482	0.952	0.227
	2006/10/21	1.66-2.68	12.146	12.209	1.000	0.245
		4.08-8.39	9.950	9.806	0.985	0.252
	2006/11/18 -11/25	1.66-2.68	6.068	6.145	1.000	0.143
		4.08-8.39	9.332	9.209	0.987	0.207
Non-dust days	2004/10/10	1.66-2.68	0.757	0.754	0.997	0.062
		4.08-8.39	2.823	2.485	0.880	0.111
	2004/12/12	1.66-2.68	2.294	1.800	0.785	0.131
		4.08-8.39	5.408	4.968	0.919	0.153
	2005/3/12	1.66-2.68	0.577	0.059	0.102	0.028
		4.08-8.39	0.774	0.660	0.852	0.041
	2005/3/25	1.66-2.68	0.455	0.291	0.641	0.046
		4.08-8.39	0.843	0.378	0.448	0.047
	2005/10/26	1.66-2.68	3.626	3.362	0.927	0.071
		4.08-8.39	4.561	4.332	0.950	0.049

Table S7 Water uptake on the surface of dry dust and $Ca(NO_3)_2$ in dust samples collected at nearby dust source (Alashan) and downwind cities (Beijing and Qingdao) during Asian dust storm events, respectively. Uptake coefficients of water under different RHs from the dust samples during Asian dust storm and from $Ca(NO_3)_2$ were adopted from Chen et al., (2020) [40].

Sites	PM_{10} (μg/m^3)	$Ca(NO_3)_2$ (μg/m^3)	Water uptake on dry dust (μg/m^3)		Water uptake by $Ca(NO_3)_2$ in dust samples (μg/m^3)		Water uptake by $Ca(NO_3)_2$ vs dry dust	
			RH 40%	RH 60%	RH 40%	RH 60%	RH 40%	RH 60%
Alashan	563±578 (n=5)	2.1±1.4	4.1±4.2	5.4±5.5	2.2±1.4	2.9±1.8	0.76±0.45	0.77±0.45
Beijing	647±398 (n=7)	17.9±16.6	4.7±2.9	6.2±3.8	18.2±16.8	24.4±22.6	4.11±3.54	4.13±3.56
Qingdao	397±333 (n=3)	6.8±4.0	2.9±2.4	3.8±3.2	6.9±4.0	9.2±5.4	3.61±2.39	3.63±2.40

References:

1. Bougiatioti A, Zarmpas P, Koulouri E *et al.* Organic, elemental and water-soluble organic carbon in size segregated aerosols, in the marine boundary layer of the Eastern Mediterranean. *Atmos Environ*. 2013; **64**: 251-262. doi: https://doi.org/10.1016/j.atmosenv.2012.09.071
2. Wang GH, Zhou BH, Cheng CL *et al.* Impact of Gobi desert dust on aerosol chemistry of Xi'an, inland China during spring 2009: differences in composition and size distribution between the urban ground surface and the mountain atmosphere. *Atmos Chem Phys*. 2013; **13**(2): 819-835.
3. Wang G, Cheng C, Meng J *et al.* Field observation on secondary organic aerosols during Asian dust storm periods: Formation mechanism of oxalic acid and related compounds on dust surface. *Atmos Environ*. 2015; **113**(0): 169-176.
4. Wu F, Song N, Hu T *et al.* Surrogate atmospheric dust particles generated from dune soils in laboratory: Comparison with field measurement. *Particuology*. 2023; **72**: 29-36. doi: https://doi.org/10.1016/j.partic.2022.02.007
5. Chi JW, Li WJ, Zhang DZ *et al.* Sea salt aerosols as a reactive surface for inorganic and organic acidic gases in the Arctic troposphere. *Atmos Chem Phys*. 2015; **15**(19): 11341-11353.
6. Ghosal S, Weber PK, Laskin A. Spatially resolved chemical imaging of individual atmospheric particles using nanoscale imaging mass spectrometry: insight into particle origin and chemistry. *Anal Methods*. 2014; **6**(8): 2444-2451.
7. Pöhlker C, Wiedemann KT, Sinha B *et al.* Biogenic potassium salt particles as seeds for secondary organic aerosol in the Amazon. *Science*. 2012; **337**(6098): 1075-1078.
8. Li W, Xu L, Liu X *et al.* Air pollution–aerosol interactions produce more bioavailable iron for ocean ecosystems. *Sci Adv*. 2017; **3**(3): e1601749, 1601741-1601746.
9. Xu J, Song S, Harrison RM *et al.* An interlaboratory comparison of aerosol inorganic ion measurements by ion chromatography: implications for aerosol pH estimate. *Atmos Meas Tech*. 2020; **13**(11): 6325-6341. doi: 10.5194/amt-13-6325-2020

10. Kawamura K, Barrie LA, Toom-Sauntry D. Intercomparison of the measurements of oxalic acid in aerosols by gas chromatography and ion chromatography. *Atmos Environ*. 2010; **44**(39): 5316-5319. doi: https://doi.org/10.1016/j.atmosenv.2010.08.051
11. Wang Y, Zhao Y, Wang Y *et al.* Organosulfates in atmospheric aerosols in Shanghai, China: seasonal and interannual variability, origin, and formation mechanisms. *Atmos Chem Phys*. 2021; **21**(4): 2959-2980. doi: 10.5194/acp-21-2959-2021
12. McNeill VF, Woo JL, Kim DD *et al.* Aqueous-phase secondary organic aerosol and organosulfate formation in atmospheric aerosols: a modeling study. *Environ Sci Technol*. 2012; **46**(15): 8075-8081. doi: 10.1021/es3002986
13. Gelaro R, McCarty W, Suárez MJ *et al.* The Modern-Era Retrospective Analysis for Research and Applications, Version 2 (MERRA-2). *J Climate*. 2017; **30**(14): 5419-5454. doi: 10.1175/jcli-d-16-0758.1
14. Ito A, Sillman S, Penner JE. Effects of additional nonmethane volatile organic compounds, organic nitrates, and direct emissions of oxygenated organic species on global tropospheric chemistry. *J Geophys Res*. 2007; **112**(D6). doi: https://doi.org/10.1029/2005JD006556
15. Sillman S, Marsik FJ, Al-Wali KI *et al.* Reactive mercury in the troposphere: Model formation and results for Florida, the northeastern United States, and the Atlantic Ocean. *J Geophys Res*. 2007; **112**(D23). doi: https://doi.org/10.1029/2006JD008227
16. Ito A, Lin G, Penner JE. Global modeling study of soluble organic nitrogen from open biomass burning. *Atmos Environ*. 2015; **121**: 103-112. doi: https://doi.org/10.1016/j.atmosenv.2015.01.031
17. Lin G, Sillman S, Penner JE *et al.* Global modeling of SOA: the use of different mechanisms for aqueous-phase formation. *Atmos Chem Phys*. 2014; **14**(11): 5451-5475. doi: 10.5194/acp-14-5451-2014
18. Ito A, Miyakawa T. Aerosol iron from metal production as a secondary source of bioaccessible iron. *Environ Sci Tech*. 2023; **57**(10): 4091-4100.
19. Feng Y, Penner JE. Global modeling of nitrate and ammonium: Interaction of aerosols and tropospheric chemistry. *J Geophys Res*. 2007; **112**(D1).
20. Ito A, Lin G, Penner JE. Reconciling modeled and observed atmospheric deposition of soluble organic nitrogen at coastal locations. *Global Biogeochem Cy*. 2014; **28**(6): 617-630. doi: https://doi.org/10.1002/2013GB004721
21. Ervens B, Turpin BJ, Weber RJ. Secondary organic aerosol formation in cloud droplets and aqueous particles (aqSOA): a review of laboratory, field and model studies. *Atmos Chem Phys*. 2011; **11**(21): 11069-11102. doi: 10.5194/acp-11-11069-2011
22. Ervens B, Volkamer R. Glyoxal processing by aerosol multiphase chemistry: towards a kinetic modeling framework of secondary organic aerosol formation in aqueous particles. *Atmos Chem Phys*. 2010; **10**(17): 8219-8244. doi: 10.5194/acp-10-8219-2010
23. Sumner AJ, Woo JL, McNeill VF. Model Analysis of Secondary Organic Aerosol Formation by Glyoxal in Laboratory Studies: The Case for Photoenhanced Chemistry. *Environ Sci Atmos*. 2014; **48**(20): 11919-11925. doi: 10.1021/es502020j
24. Waxman EM, Dzepina K, Ervens B *et al.* Secondary organic aerosol formation from semi- and intermediate-volatility organic compounds and glyoxal: Relevance of O/C as a tracer for aqueous multiphase chemistry. *Geophy Res Lett*. 2013; **40**(5): 978-982. doi: https://doi.org/10.1002/grl.50203
25. Zogka AG, Lostier A, Papadimitriou VC *et al.* Unraveling the Uptake of Glyoxal on a Diversity of Natural Dusts and Surrogates: Linking Dust Composition to Glyoxal Uptake and Estimation of Atmospheric Lifetimes. *ACS Earth Space Chem*. 2024; **8**(6): 1165-1178. doi: 10.1021/acsearthspacechem.3c00359

26. Xu J, Zhang X, Zhao W *et al.* High-resolution physicochemical dataset of atmospheric aerosols over the Tibetan Plateau and its surroundings. *Earth Syst Sci Data*. 2024; **16**(4): 1875-1900. doi: 10.5194/essd-16-1875-2024
27. Hanson DR, Ravishankara AR, Solomon S. Heterogeneous reactions in sulfuric acid aerosols: A framework for model calculations. *J Geophys Res*. 1994; **99**(D2): 3615-3629. doi: https://doi.org/10.1029/93JD02932
28. Curry LA, Tsui WG, McNeill VF. Technical note: Updated parameterization of the reactive uptake of glyoxal and methylglyoxal by atmospheric aerosols and cloud droplets. *Atmos Chem Phys*. 2018; **18**(13): 9823-9830. doi: 10.5194/acp-18-9823-2018
29. Gaston CJ, Riedel TP, Zhang Z *et al.* Reactive Uptake of an Isoprene-Derived Epoxydiol to Submicron Aerosol Particles. *Environ Sci Technol*. 2014; **48**(19): 11178-11186. doi: 10.1021/es5034266
30. V. Buxton G, N. Malone T, Arthur Salmon G. Oxidation of glyoxal initiated by OH in oxygenated aqueous solution. *J Chem Soc Faraday trans*. 1997; **93**(16): 2889-2891. doi: 10.1039/A701468F
31. Schaefer T, Schindelka J, Hoffmann D *et al.* Laboratory Kinetic and Mechanistic Studies on the OH-Initiated Oxidation of Acetone in Aqueous Solution. *J Phys Chem A*. 2012; **116**(24): 6317-6326. doi: 10.1021/jp2120753
32. Eddingsaas NC, VanderVelde DG, Wennberg PO. Kinetics and Products of the Acid-Catalyzed Ring-Opening of Atmospherically Relevant Butyl Epoxy Alcohols. *J Phys Chem A*. 2010; **114**(31): 8106-8113. doi: 10.1021/jp103907c
33. Marais EA, Jacob DJ, Jimenez JL *et al.* Aqueous-phase mechanism for secondary organic aerosol formation from isoprene: application to the Southeast United States and co-benefit of SO(2) emission controls. *Atmos Chem Phys*. 2016; **16**(3): 1603-1618. doi: 10.5194/acp-16-1603-2016
34. Kampf CJ, Waxman EM, Slowik JG *et al.* Effective Henry's Law Partitioning and the Salting Constant of Glyoxal in Aerosols Containing Sulfate. *Atmos Chem Phys*. 2013; **47**(9): 4236-4244. doi: 10.1021/es400083d
35. Waxman EM, Elm J, Kurtén T *et al.* Glyoxal and Methylglyoxal Setschenow Salting Constants in Sulfate, Nitrate, and Chloride Solutions: Measurements and Gibbs Energies. *Environ Sci Technol*. 2015; **49**(19): 11500-11508. doi: 10.1021/acs.est.5b02782
36. Betterton EA, Hoffmann MR. Henry's law constants of some environmentally important aldehydes. *Environ Sci Atmos*. 1988; **22**(12): 1415-1418. doi: 10.1021/es00177a004
37. Cole-Filipiak NC, O'Connor AE, Elrod MJ. Kinetics of the hydrolysis of atmospherically relevant isoprene-derived hydroxy epoxides. *Environ Sci Atmos*. 2010; **44 17**: 6718-6723.
38. Peng C, Chan MN, Chan CK. The Hygroscopic Properties of Dicarboxylic and Multifunctional Acids: Measurements and UNIFAC Predictions. *Environ Sci Technol*. 2001; **35**(22): 4495-4501. doi: 10.1021/es0107531
39. Shi Z, Zhang D, Hayashi M *et al.* Influences of sulfate and nitrate on the hygroscopic behaviour of coarse dust particles. *Atmos Environ*. 2008; **42**(4): 822-827.
40. Chen L, Peng C, Gu W *et al.* On mineral dust aerosol hygroscopicity. *Atmos Chem Phys*. 2020; **20**(21): 13611-13626. doi: 10.5194/acp-20-13611-2020
